# A network-driven computational framework for identifying FDA-approved drug repurposing across heterogeneous brain cancers

**DOI:** 10.3389/fmolb.2026.1768081

**Published:** 2026-02-17

**Authors:** Om Prakash

**Affiliations:** The Institute of Mathematical Sciences (IMSc), Chennai, India

**Keywords:** brain, cancer, drug, Food and Drug Administration, network, repurposing

## Abstract

**Background:**

Brain cancers are notorious for their heterogeneity, which complicates therapeutic decisions because of recurrently dysregulated signaling pathways. Cancer system characterizations have allowed the identification of some key components involved in brain cancer, such as the epidermal growth factor receptor, *BRAF*, platelet-derived growth factor receptor alpha, *TP53*, O6-methylguanine-DNA methyltransferase, cyclin-dependent kinase 1/2/3/4, cyclooxygenase 1/2, vascular endothelial growth factor receptor 2, telomerase reverse transcriptase, and CYP2D6, along with the U87 cell line. These components are the core focus of protocol designs for rational drug selection. For drug repurposing, these designed protocols are generally hypothesized with Food and Drug Administration (FDA)-approved drugs.

**Methods:**

In the present study, a protocol was designed to address this complexity using the identified pathway components. These components served as the basis for defining the signatures of small molecules. The set of molecular signatures was then used to develop a network-driven computational framework. Accordingly, two in-house applications were developed, namely, a molecular profile generator called “*in-mac*” and a network-based database called “ReBrain”, derived from FDA-approved drug molecules. *In-mac* is a computational bioassay platform that generates the activity profiles of small molecules, while ReBrain is a database used for broad-spectrum drug-repurposing analytics. The performance of the profile set was evaluated and validated using five machine-learning models with three different classified datasets.

**Results:**

A total of 2,809 FDA-approved drug molecules (molecular weight ≤500 Da) were profiled using *in-mac*, and each molecular profile included fifteen-dimensional activity signatures. The profile set was then proven to have significant potential for drug repurposing. The molecular profiles were next used in a regression analysis, followed by the calculation of the intermolecular Euclidean distances and the development of an intermolecular network. The ReBrain platform also enabled *in silico* knockout or knock-in capabilities for specific pathway components. Finally, network refinement was achieved using the molecular weights and distance thresholds.

**Conclusion:**

The proposed profile-network-based method achieved 70%–95% accuracy for drug repurposing across different disease categories related to the brain. *In-mac* and ReBrain were used for the repurposing of known drugs for the treatment of brain cancer. As a result, three repurposed drugs were identified as priorities: (i) mefloquine (reference drug: vorasidenib citrate), (ii) clofibric acid (reference drug: carmustine), and armillarisin A (reference drug: lomustine). These results also suggest repurposing candidates for synergistic combinations across different brain tumors. The two applications developed in this work are freely accessible and in the public domain at https://assay.smallmoles.com/escorwin.

## 1 Introduction

Brain cancers are considered high-value research targets because of the complex dysregulation of multiple signaling pathways that leads to tumor growth, survival, and resistance. Commonly known pathways include those of receptor tyrosine kinase (RTK), epidermal growth factor receptors (EGFRs), platelet-derived growth factor receptor alpha (PDGFRA) (Li et al., 2023; Stachyra and Grzybowska-Szatkowska, 2025), MAPK with *BRAF* mutations (Talloa et al., 2022), reorganization of cell-cycle regulation of cyclin-dependent kinases (CDK1/2/3/4) along with cell-cycle checkpoints (Li et al., 2023), DNA repair and apoptotic systems (*TP53* and O6-methylguanine-DNA methyltransferase (MGMT)) (Yu et al., 2020; Donson et al., 2007), telomere maintenance mechanisms such as telomerase reverse transcriptase (TERT) (Patel et al., 2020; Killela et al., 2013), and inflammatory mediators and angiogenesis-related signaling (Li et al., 2023). Of these, researchers have explored the specific roles of cyclooxygenases (COX-1/COX-2) and CYP2D6 in brain tumors, including their inflammatory and metabolic processes, which are well known in cancer biology. The direct involvement of these dysregulated pathways in the treatment of brain cancers makes them attractive candidates for drug repurposing. There has been an increased interest in drug repurposing in recent times owing to the time and cost savings that can be achieved compared to developing an entirely new drug. Among these, molecules approved by the Food and Drug Administration (FDA) are at the forefront of drug repurposing because of their established safety profiles, which can accelerate clinical translation (Xia et al., 2024). Brain-cancer-related drug repurposing requires additional attention to the ability of a drug to penetrate the blood–brain barrier (BBB) so that it can be delivered effectively to the central nervous system (CNS) (Ntafoulis et al., 2022).

The strategy for drug repurposing requires observation of the multidimensional interplay between molecular profiles and network-driven methodologies. Networks can be derived from computational models, may be real biological networks, or may be defined based on systems biology approaches (Gonzalez et al., 2025). However, despite the integration of multiple approaches, drug repurposing is scientifically and clinically limited by the need to adopt advanced methodologies against barriers, such as pharmacological boundaries, clinical trial parameters, and molecular stratification (Xia et al., 2024). In this context, we hypothesize that FDA-approved drugs can be used to observe dysregulated signaling pathways and can enable the exploration of therapeutically meaningful strategies for drug repurposing.

Network graphs are often used to capture the complexity of a biological system with isolated components relevant to the purpose of the study. These studies aim to understand the perturbations existing in a network of targets, genes, expressions, etc. The graph approach has also been adopted in different areas, such as network medicine and network pharmacology, in support of rational drug discovery (Joshi et al., 2025). Network analysis is also known for efficient drug repurposing as it integrates paired node data (Selvaraj et al., 2022). Beyond these uses, network analysis can be employed to reveal hidden parameter combinations from multiple biological pathways (Li et al., 2016).

Several therapeutic agents targeting brain cancers have received FDA approval for neurooncological purposes. Some of the well-known drugs include temozolomide for standard chemoradiation therapy, lomustine and carmustine for high-grade gliomas, and bevacizumab as a monoclonal antibody targeting vascular endothelial growth factors (Buzatu et al., 2025). Temozolomide is also used in combination therapies (Fisher and Adamson, 2021). Several mitochondrial inhibitors, such as trifluoperazine, mitoxantrone, and pyrvinium pamoate, have been repurposed for the treatment of brain cancers. Multitarget antiangiogenesis drugs such as axitinib, sorafenib, lenvatinib, sunitinib, cabozantinib, and regorafenib have also been approved for brain cancer therapy (Buzatu et al., 2025).

Because of the frequent chaotic interactions among altered targets, such as EGFRs, *BRAF*, PDGFRA, TP53, MGMT, CDKs, COX-1/2, vascular endothelial growth factor receptor 2 (VEGFR2), TERT, and CYP2D6, drug repurposing poses a high degree of uncertainty for therapeutic decision-making. However, the ensemble packaging of molecular signatures (or molecular profiles) in reference to these chaotic interactions reveals patterns for drug repurposing. This motivated us to develop two applications using molecular signatures, namely, “*in-mac*” and “ReBrain.” These applications were designed to reduce the complexity of the processes required for drug repurposing. Accordingly, scientifically simple observations were documented by generating structured molecular profiles that mirror disrupted pathways and were compared with FDA-approved drugs in an unbiased and data-driven manner. Together, these applications provide a systematic framework that supports more confident reasoning and discovery in drug repurposing. The developed applications were grounded in the need to analyze small molecules against the key dysregulated components of brain cancer and to organize the resulting data in a systematic platform for repurposing therapeutics. Thus, a computational profiling system was created to capture characteristic drug patterns in relation to different pathway components, and a database was developed to catalog these profiles for all FDA-approved drugs. Together, these resources enable large-scale comparisons of drug behaviors and the construction of a flexible network to identify repurposing opportunities. Finally, existing drugs for the treatment of brain cancer were processed through *in-mac* and ReBrain to discover the new repurposed drugs.

The molecular profile capabilities can be evaluated and validated through the consistent accuracy obtained using multiple machine-learning models, which are considered rigorous methods. Obtaining consistent accuracies for multiple machine-learning models also proves that the corresponding molecular profiles can be used for data featurization. The machine learning methods at the forefront of these efforts include logistic classification, decision trees, random forests, artificial neural networks, and support vector machines. These methods have already demonstrated strong performances for clinical predictions (Wu et al., 2023; Nwanosike et al., 2022). Similarly, machine-learning and deep-learning methods are well known in the fields of oncology and precision medicine for combining the molecular and phenotypic profiles for various data-driven validation frameworks applicable across multiple domains (Gawri et al., 2024; Balamurugan et al., 2024a; Jayaraman et al., 2024; Balamurugan et al., 2024b; Balamurugan et al., 2024c).

The proposed integrative approach allows prioritization of candidate repurposed drugs in reference to any chosen molecule to facilitate detailed exploration of their pathway contributions. The top-priority repurposed agents can be further reevaluated through *in-mac*-based analysis. Collectively, the proposed pathway-dysregulation-profile-based network-driven framework provides a powerful strategy to accelerate the discovery of repurposed, FDA-approved drugs.

## 2 Materials and methods

**FIGURE 1:**
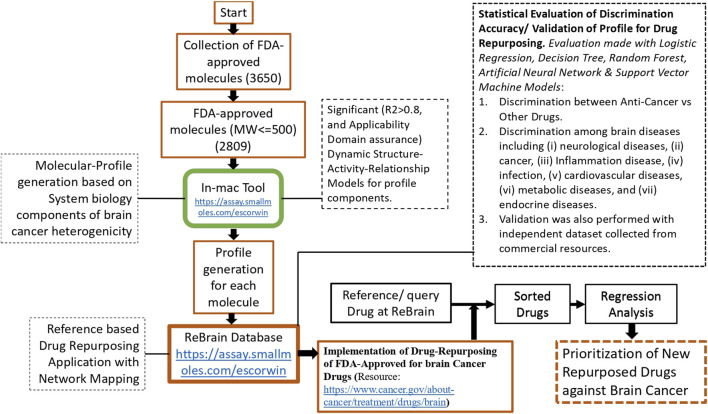
Schematic illustration of the workflow of this study. The workflow starts with the development of the ReBrain application, based on molecular profiles generated for FDA-approved drugs. These molecular profiles were defined to encompass the heterogeneity of brain cancers. Each profile component was derived using the significant structure–activity relationship model of each drug. The efficiency of each molecular profile was evaluated using five machine-learning models, and the performance was evaluated for discrimination of anti-cancer vs. other drugs. The performance was also evaluated to discriminate among brain diseases, including (i) neurological diseases, (ii) cancers, (iii) inflammatory diseases, (iv) infections, (v) cardiovascular diseases, (vi) metabolic diseases, and (vii) endocrine diseases. The findings were validated using an independent dataset collected from commercial resources. The ReBrain database is a network-based tool for reference-based drug-repurposing applications.

The methodological rationale of this study is that brain cancers exhibit high molecular heterogeneity involving multiple signaling pathways, such that single targets or linear models are insufficient to capture the resulting biological interactions; therefore, a network-driven computational framework needs to be adopted. Molecular-signature-based profiling is significant for capturing patterns from multidimensional pathway interactions. The repurposed drugs were chosen from FDA-approved compounds because their established safety profiles could reduce the time and costs associated with drug development. Additionally, emphasis was placed on considering the BBB to ensure the clinical relevance of deliverability to the CNS. Therefore, existing brain cancer drugs were used in the analysis. Collectively, this integrative systems biology approach provides an informed method for drug repurposing. Therefore, an integrated computational workflow was developed by combining pathway-level dysregulation profiling and network-based searching of repurposed drugs based on reference drugs to systematically identify FDA-approved drugs with repurposing potential for brain cancers. Central to this workflow, we developed two new web-based applications called *in-mac* and ReBrain. The following sections describe the datasets used, application descriptions, and implementation for drug repurposing, which enable the identification and prioritization of candidate drugs based on their mechanistic relevance and pathway-level perturbation profiles (Figure 1).

### 2.1 FDA-approved drugs

To ensure broad chemical and pharmacological diversity in the data, we comprehensively considered 2,809 FDA-approved small-molecule drugs for repurposing. Here, the targets were restricted to drug molecules with molecular weights ≤500 Da.

### 2.2 Brain-cancer-related pathway components

To consider the neurooncogenic pathways in brain cancer, 15 key molecular components were used for drug profiling. These components included cell lines (U87 glioblastoma), oncogenes and receptors (*BRAF*, EGFR, and PDGFRA), tumor suppressors and DNA repair enzymes (TP53 and MGMT), cell-cycle regulators (CDK1, CDK2, CDK3, and CDK4), inflammatory mediators (COX-1 and COX-2), an angiogenesis-related kinase inhibitor (VEGFR2 kinase inhibitor), a telomere maintenance factor (TERT), and a drug metabolism enzyme (CYP2D6). These components were used as the environments for the computational bioassay panel to generate the molecular profiles.

### 2.3 *In-mac* application for generating drug molecular profiles

To reflect the effects of a drug molecule on brain-cancer-related components, the *in-mac* application integrated computational bioassay environments for the 15 components noted above. The drug profile consisted of a set of multidimensional signatures derived from computational bioassays. These signatures are quantitatively weighted in accordance with the structure–activity relationships (SARs) of each of the molecules. The i*n-mac* application utilized the Monte–Carlo method to process the small molecules against each bioassay environment to extract the signatures. The JSME Editor (Bienfait et al., 2013) was used to receive inputs into *in-mac*, and any experiment could be replicated using the “undo” and “redo” functions.

#### 2.3.1 Validation of the profile-based method and evaluation of framework robustness for different diseases and datasets for network-based drug repurposing

Profiles generated for the FDA-approved drugs were processed to evaluate their discrimination accuracies and validate their drug-repurposing utilities. The curated dataset was categorized into anti-cancer and non-cancer (other) drugs and was processed for the discrimination accuracy. For disease-specific analysis, the curated dataset was further classified into seven major disease categories as follows: (i) neurological diseases, (ii) cancers, (iii) inflammatory diseases, (iv) infectious diseases, (v) cardiovascular diseases, (vi) metabolic diseases, and (vii) endocrine diseases. All datasets were preprocessed prior to model training and further processed before model evaluation. All datasets and scripts used in this work are available in the public domain at the following link: https://github.com/omprakashdskpdf/inmacrebrain.git.

To evaluate the discrimination capabilities of the drug profiles, multiple machine learning methods were implemented, including logistic regression, decision tree, random forest, artificial neural network, and support vector machine. Each model was trained using the same dataset to ensure comparability.

Three discrimination evaluations were then performed (Li et al., 2023) as follows. In the discrimination of anti-cancer vs. other drugs, the dataset of 2,809 molecules was grouped into two classes (Stachyra and Grzybowska-Szatkowska, 2025). For disease category discrimination, multiclass classification modeling was performed with drugs known to act on the brain, based on the seven classes noted above (Talloa et al., 2022). Finally, external validation was performed to evaluate the robustness of the profile-based protocol using an independent dataset (1,392 molecules) collected from commercial resources. Here, three class datasets were collected for antiangiogenesis (276 molecules), antiviral (562 molecules), and anticardiovascular (554 molecules) activities.

Comparative statistical evaluations were performed to assess the discrimination accuracy using receiver operating characteristic (ROC) plots and their area under the curve (AUC) values. A default threshold of 0.5 was applied for binary classification. For multiclass classification, each class was assessed in reference to the other classes in the combined set. During model development, the hyperparameters were optimized using a grid search strategy within the training dataset. To handle class imbalances in both binary and multiclass classification modeling, stratified sampling was considered, wherein each class was evaluated relative to the other classes combined.

The mathematical indicators for evaluating the classification model are as follows:

A confusion matrix with true-positive (TP), true-negative (TN), false-positive (FP), and false-negative (FN) elements. The performance metrics are defined as follows:

Accuracy = (TP + TN)/(TP + FP + TN + FN)

Sensitivity = TP rate (TPR) = Recall = TP/(TP + FN).

Specificity = TN/(TN + FP).

Precision = TP/(TP + FP).

F1-score = 2 × Precision × Recall/(Precision + Recall).

ROC plots for TPR vs. FP rate (FPR)

FPR = 1 – Specificity.

The AUC for the ROC is given as

$$AUC=\int_{0}^{1}TPR\left(FPR\right)d\left(FPR\right).$$

#### 2.3.2 Functional benchmarking with state-of-the-art drug-repurposing tools

Functional benchmarking was performed in terms of selectivity toward brain diseases, the ability to search for repurposed drugs based on profile signatures and repurposing with a reference drug, and the availability of a prioritized list. Four web-based applications were used for comparisons in this step: (i) drug-repurposing hub (Broad Institute; https://clue.io/repurposing), (ii) DisGeNET (https://www.disgenet.org/), (iii) Hetionet/Neo4j drug repurposing graph (https://het.io/), and (iv) Open Targets Platform (https://platform.opentargets.org/).

### 2.4 ReBrain database for finding functionally similar drugs based on brain cancer profiles

FDA-approved drugs were processed through *In-Mac* to prepare their molecular profiles. These profiles were then used to develop a broad-spectrum drug-repurposing database called ReBrain that includes a curated set of 2,809 FDA-approved drugs with molecular weights ≤500 Da. ReBrain offers multiple features, including the ability to process reference query drug can against each FDA-approved molecule for drug–drug profile regression and Euclidean distance metric calculation. These distance metrics are used to construct a dynamic drug-repurposing network based on a user query. A new set of processes is implemented for repurposing for each queried drug. The database dynamicity can be visualized at the client end, and the network can be refined by molecular weight, target involvement (by knock-in and knockout), and similarity thresholds. Although ReBrain was developed as a complementary platform to support broad-spectrum drug-repurposing analytics involving FDA-approved drug molecules with brain-cancer-related components, it can also be used for other target profile combinations.

### 2.5 Drug–drug profile regression and network construction

Regression-based weight matrices were used to assess the relationships between drug pairs. These matrices included functional similarity for drug repurposing and influenced the relationships between drug pairs. Furthermore, the obtained weight values were used to calculate the Euclidean distances to construct a drug-repurposing network, in which the nodes represent drugs and the inverse lengths of the edges reflect the functional similarities or distances between the nodes. These networks can be regenerated by selecting or searching for reference drugs as queries. To refine the search space for repurposing candidates in the resulting network, sliders are available for adjusting the molecular weight and Euclidean distance thresholds. These thresholds were included to balance the load of network data. Additionally, knock-in and knockout operations can be performed on individual profile components through checkboxes. To aid selectivity, specific molecular assay components can be included or excluded to tailor the network analysis for specific hypotheses or experimental conditions. Thus, detailed drug-target information, including the interaction weights and pathway contributions, can be displayed alongside each node. The top-priority results of the analysis can be considered repurposed drugs and may be reevaluated against brain cancers. Furthermore, the selected top-priority repurposed drugs can be cross-referenced with existing literature to find the necessary experimental or clinical evidence supporting their potential efficacy in the context of brain cancer.

### 2.6 Finding new repurposed drugs against brain cancer

To find new repurposed drugs for brain cancer treatment, existing small-molecule drugs (from https://www.cancer.gov) were used as the reference drugs. The results were further analyzed for validation, molecular interactions, and the ability to cross the BBB.

### 2.7 Molecular interactions of the finalized repurposed drugs

The molecular interactions of the identified repurposed drugs were assessed using AutoDock Vina. The target protein considered in this step was isocitrate dehydrogenase 1/2 (IDH1/2) (PDB: 6U4J), and the ligands used were vorasidenib citrate (reference) and mefloquine (repurposed drug). These drug molecules were also evaluated for performance at the BBB.

## 3 Results

Using the integrated computational pipeline and the two newly developed platforms, a systematic profiling was performed on the FDA-approved drug molecules. This combined framework enabled the generation of high-resolution molecular profiles, identification of recurrent pathway perturbations, and construction of a comprehensive drug–drug similarity network for drug repurposing. The results of these efforts are highlighted in three stages as follows: pathway-level alterations revealed by *in-mac*, network architecture and drug–drug functional similarities identified through ReBrain, and prioritization of candidate FDA-approved molecules for drug repurposing.

### 3.1 Profiles for FDA-approved drugs (based on *in-mac*)

**FIGURE 2:**
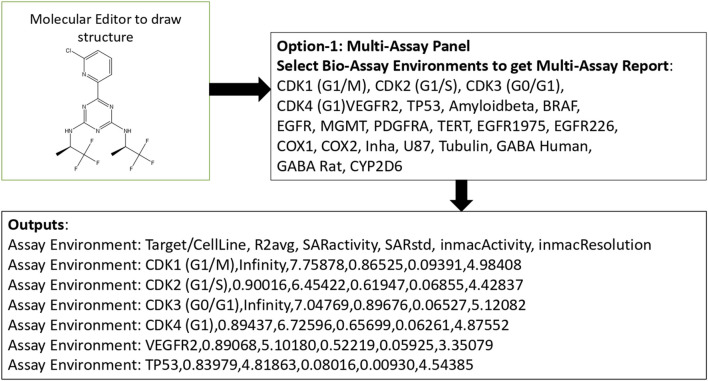
Pictorial representation of the front-end components of *in-mac* comprising three parts: a molecular editor to draw the query structure, a multiassay panel to select the profile components, and an output panel to display the selected profile components. The outputs include the average regression coefficient (R2avg), the predicted pChEMBL value (SARactivity), the standard deviation of the SARactivity (SARstd), the weight of the predicted activity (inmacActivity), and the resolution of the prediction (inmacResolution). SARactivity, inmacActivity, and inmacResolution are considered the primary components of a molecular profile.

The *in-mac* computational bioassay platform simulated the *in vitro* experimental processes of FDA-approved small molecules through a SAR-based approach. This step generated the molecular profiles of the drugs with multiassay components related to brain cancer, including cell lines (U87), oncogenes (*BRAF*, *EGFR*, and *PDGFRA*), tumor suppressors (TP53 and MGMT), cell-cycle regulators (CDK1–4), inflammatory enzymes (COX-1/2), angiogenesis kinases (VEGFR2 kinase inhibitor), the telomere maintenance factor (TERT), and metabolic enzymes (CYP2D6). Here, *in-mac* received the structures of the drug molecules along with the assay environments defined under “multiassay panel” and provided outputs against each of the assay environments. These outputs included the average regression coefficient as *R2avg*, normalized predicted activity as *SARactivity*, standard deviation against *SARactivity* as *SARstd*, *in silico* activity value as *inmacActivity*, and inverse of the model bias as *inmacResolution*. For drug repurposing, *inmacActivity* was used as the key element of the molecular profile. Fifteen such *inmacActivity* values from the computational bioassays collectively contributed to the molecular profiles. The *inmacActivity* values were applied using the *in-mac* platform to successfully generate the molecular profiles of the 2,809 FDA-approved small-molecule drugs of molecular weights ≤500 Da. The molecular profiles were used to develop a network-graph-based database in the next step for drug repurposing (Figure 2).

#### 3.1.1 Evaluation of the discrimination performance of the profiles used with ReBrain

The drug profile capabilities were evaluated using different machine-learning methods, namely, logistic regression, decision tree, random forest, artificial neural network, and support vector machine. The model performances reflected the patterns of the generated profile capabilities.

Three discrimination evaluations were performed for each machine-learning method (Li et al., 2023), namely, anti-cancer vs. other drugs as a binary classification modeling (Stachyra and Grzybowska-Szatkowska, 2025), disease category discrimination based on seven-class classification modeling (Talloa et al., 2022), and validation with an independent dataset as a three-class classification modeling. The statistical evaluations reflecting the profile validation for drug repurposing are available in our GitHub repository (*GitHub: Supplementary_Data_Evaluation_Validation.zip*).

##### 3.1.1.1 Discrimination of anti-cancer vs. non-cancer molecules

The five classification models were evaluated with the same dataset using 15 profile components along with the 2,809 drug samples encompassing two classes of data, i.e., anti-cancer and non-cancer molecules. The system profile was able to successfully help classify approximately all the samples. Multiple classification methods showed similar results, which excluded the possibility of overfitting; the statistical summary of each model is shown in Table 1. The ROC plots are shown in the supplementary data, and their AUC values were found to reach the ideal prediction of >99% (Table 1).

**TABLE 1:** Statistical credentials of the five machine-learning models for the binary classification of drugs as anti-cancer or non-cancer molecules based on the evaluation of accuracy, weighted precision, recall, F1-score, and area under the curve (AUC). The results show overall high predictive performances for all models.

Model	Accuracy	Precision (weighted)	Recall (weighted)	F1-score (weighted)	AUC score
Logistic regression	0.996441	0.996455	0.996441	0.996376	0.999931
Decision tree	1	1	1	1	1
Random forest	1	1	1	1	1
Support vector machine	0.998221	0.998224	0.998221	0.998205	1
Artificial neural network	1	1	1	1	1

##### 3.1.1.2 Discrimination of seven disease classes related to the brain

**FIGURE 3:**
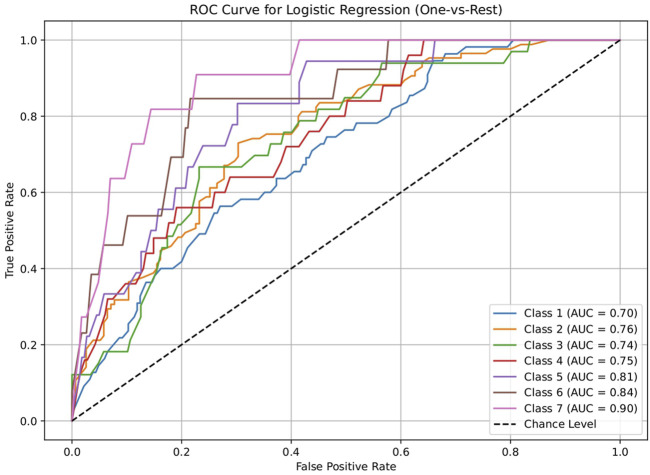
Receiver operating characteristics (ROC) plot of the logistic regression model for discriminating seven classes of brain-related diseases. The disease classes are neurological (class 1), cancer (class 2), inflammatory (class 3), infectious (class 4), cardiovascular (class 5), metabolic (class 6), and endocrine (class 7). The classification performance of the model was significant, indicating the ability of the generated molecular profile for network-based drug repurposing.

**FIGURE 4:**
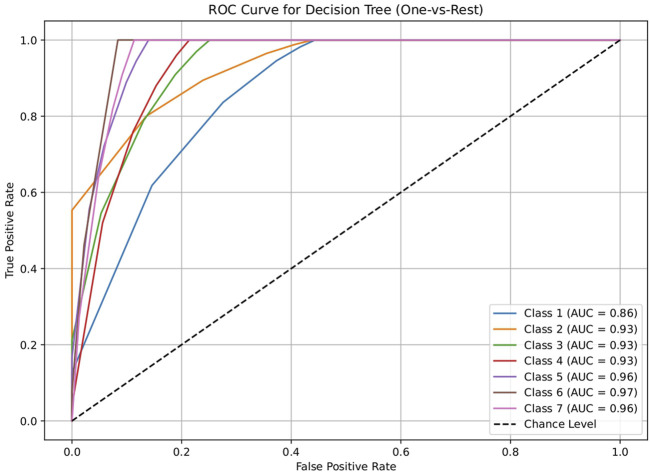
ROC plot of the decision tree model for discriminating seven classes of brain-related diseases. The disease classes were neurological (class 1), cancer (class 2), inflammatory (class 3), infectious (class 4), cardiovascular (class 5), metabolic (class 6), and endocrine (class 7). The classification performance of the model was significant, indicating its ability to generate molecular profiles for network-based drug repurposing.

**FIGURE 5:**
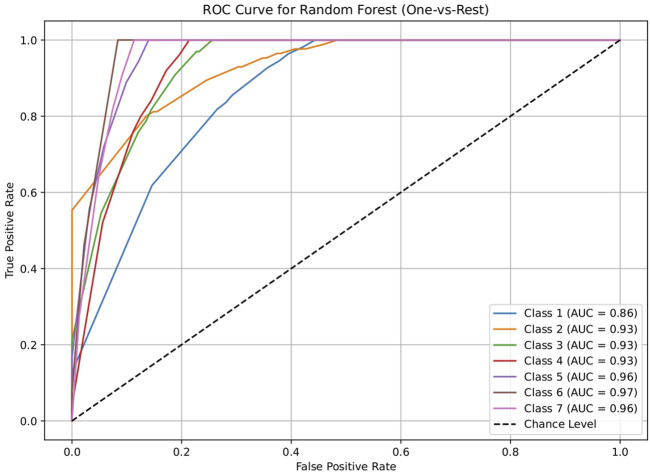
ROC plot of the random forest model for discriminating seven classes of brain-related diseases. The disease classes were neurological (class 1), cancer (class 2), inflammatory (class 3), infectious (class 4), cardiovascular (class 5), metabolic (class 6), and endocrine (class 7). The classification performance of the model was significant, indicating its ability to generate molecular profiles for network-based drug repurposing.

**FIGURE 6:**
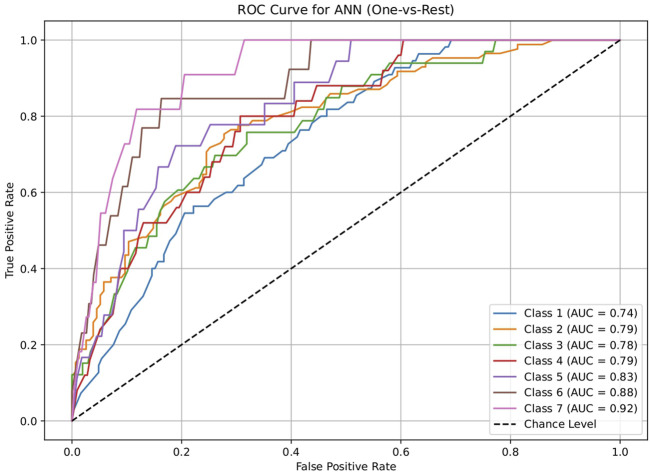
ROC plot of the artificial neural network (ANN) model for discriminating seven classes of brain-related diseases. The disease classes were neurological (class 1), cancer (class 2), inflammatory (class 3), infectious (class 4), cardiovascular (class 5), metabolic (class 6), and endocrine (class 7). The classification performance of the model was significant, indicating its ability to generate molecular profiles for network-based drug repurposing.

**FIGURE 7:**
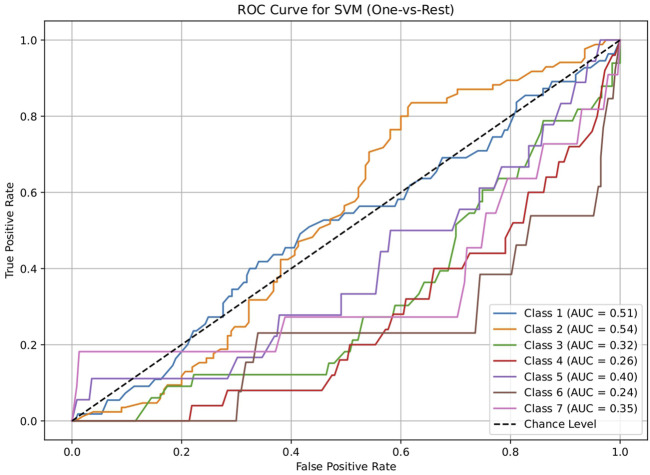
ROC plot of the support vector machine (SVM) model for discriminating seven classes of brain-related diseases. The disease classes were neurological (class 1), cancer (class 2), inflammatory (class 3), infectious (class 4), cardiovascular (class 5), metabolic (class 6), and endocrine (class 7). The classification performance of the model was not significant.

The dataset of 2,809 molecules was further processed to extract molecules with targeted brain activity; these molecules were then grouped into seven disease classes related to the brain, namely, neurological (class 1), cancer (class 2), inflammatory (class 3), infectious (class 4), cardiovascular (class 5), metabolic (class 6), and endocrine (class 7). Each class was evaluated against the remaining classes to quantify the ROC and AUC (Figures 3–7). As shown in Table 2, except for the support vector machine, all methods showed significant performance, with AUC values ranging between 0.70 and 0.95; this indicates that the generated profiles are suitable for drug repurposing.

**TABLE 2:** AUC values of the five machine-learning models investigated in this study (logistic regression, decision tree, random forest, artificial neural network, and support vector machine). These models were used to evaluate the discriminating capacities for seven disease classes. Each class was assessed using a one vs. rest approach. The results show that all methods except the support vector machine performed significantly, suggesting the drug repurposing capabilities of the molecular profiles.

Model	Class 1	Class 2	Class 3	Class 4	Class 5	Class 6	Class 7
	Neurological vs. rest	Cancer vs. rest	Inflammation vs. rest	Infection vs. rest	Cardiovascular vs. rest	Metabolic vs. rest	Endocrine vs. rest
Logistic regression	0.70034398	0.755294118	0.743522178	0.752186047	0.812187187	0.84259573	0.898769353
Decision tree	0.860638821	0.930777989	0.929732104	0.928744186	0.960085085	0.967299221	0.959110758
Random forest	0.86034398	0.928045541	0.929439321	0.928186047	0.959834835	0.967299221	0.959110758
Artificial neural network	0.741818182	0.788614801	0.778363344	0.793860465	0.830955956	0.884615385	0.915045653
Support vector machine	0.505552826	0.536394687	0.320158103	0.26455814	0.396521522	0.23737716	0.35371179

##### 3.1.1.3 Validation with an independent dataset

**FIGURE 8:**
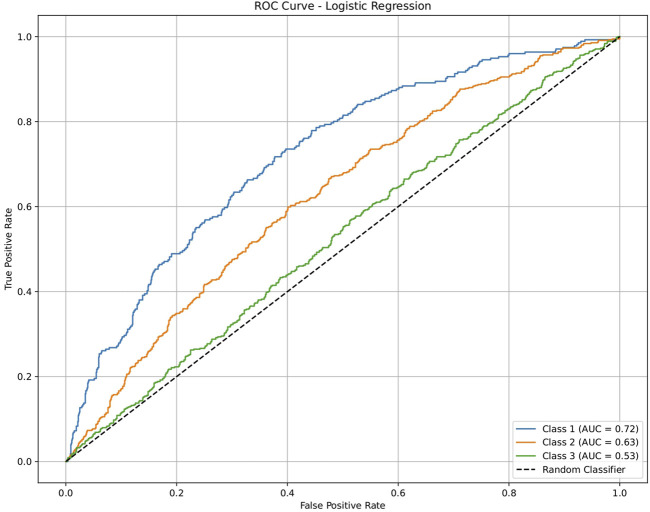
ROC plot of the logistic regression model for discriminating three classes of drugs in an independent dataset. Class 1: antiangiogenesis molecules; class 2: antiviral molecules; class 3: anticardiovascular molecules. The highest accuracy was observed for class 1.

**FIGURE 9:**
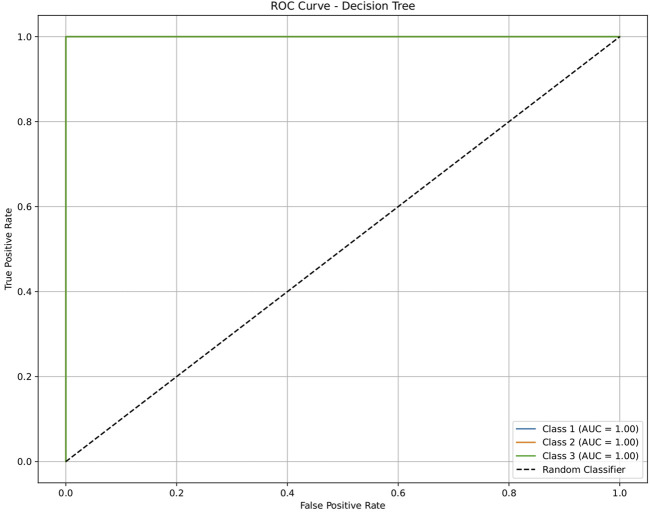
ROC plot of the decision tree model for discriminating three classes of drugs in an independent dataset. Class 1: antiangiogenesis molecules; class 2: antiviral molecules; class 3: anticardiovascular molecules. The model showed significant and similar performances for all classes.

**FIGURE 10:**
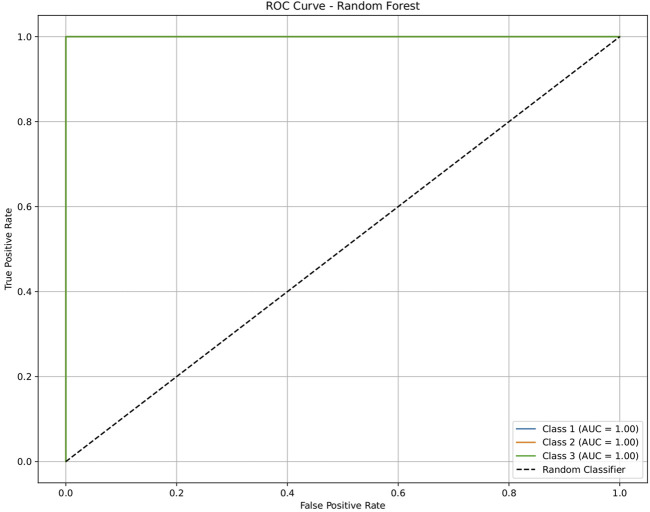
ROC plot of the random forest model for discriminating three classes of drugs in an independent dataset. Class 1: antiangiogenesis molecules; class 2: antiviral molecules; class 3: anticardiovascular molecules. The model showed significant and similar performances for all classes.

**FIGURE 11:**
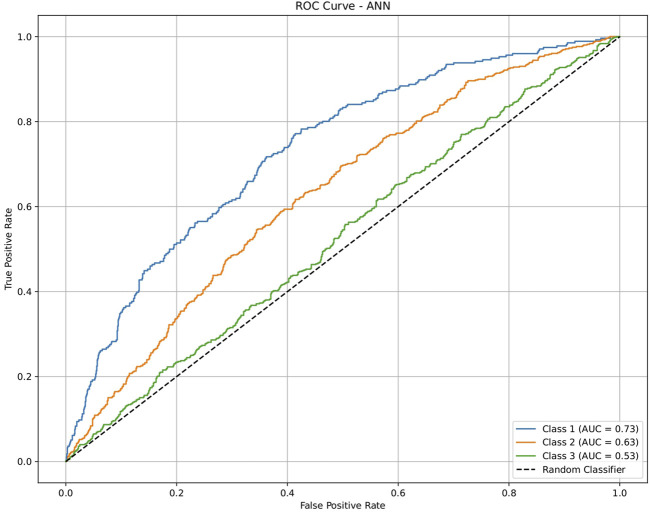
ROC plot of the ANN model for discriminating three classes of drugs in an independent dataset. Class 1: antiangiogenesis molecules; class 2: antiviral molecules; class 3: anticardiovascular molecules. The highest accuracy was observed for class 1.

**FIGURE 12:**
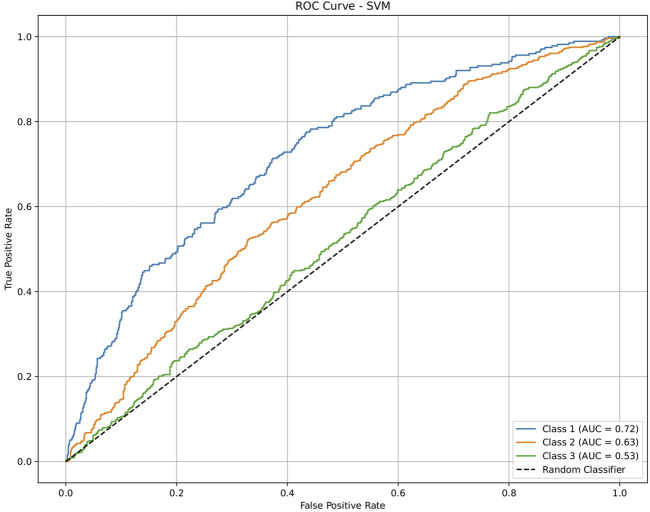
ROC plot of the SVM model for discriminating three classes of drugs in an independent dataset. Class 1: antiangiogenesis molecules; class 2: antiviral molecules; class 3: anticardiovascular molecules. The highest accuracy was observed for class 1.

To validate the ability of the molecular profile and the framework for drug repurposing, we used a generalized dataset that was not specific to the brain but contained similar disease categories for the drug activities, including antiangiogenesis (class 1), antiviral (class 2), and anticardiovascular (class 3) effects, from independent sources. Each class was evaluated against the remaining classes and quantified via ROC plots and AUC values (Figures 8–12). All machine-learning methods showed significant performances, with the AUC values ranging between 0.52 and 1.0 (Table 3); this indicates that the generated profiles are suitable for drug repurposing.

**TABLE 3:** Validation of molecular profile capabilities using five machine-learning models on an independent dataset. The dataset contained information on drug activities against three disease categories (angiogenesis, viral, and cardiovascular). The AUC values were mostly in the range of 0.52–0.73 for class discrimination.

Model	Class 1	Class 2	Class 3
	Antiangiogenesis	Antiviral	Anticardiovascular
Logistic regression	0.72220809	0.625580099	0.528746854
Decision tree	1	1	1
Random forest	1	1	1
Artificial neural network	0.732793932	0.630946825	0.528304261
Support vector machine	0.724693121	0.626118068	0.525594463

#### 3.1.2 Functional benchmarking

Functional benchmarking of *in-mac* and ReBrain was performed qualitatively using existing drug-repurposing approaches against four state-of-the-art tools. This comparison is important to demonstrate the added value and novelty of the proposed framework. Benchmarking was performed in terms of specificity for brain-related diseases, a selective search feature based on profile signatures, searching for known reference drugs, and providing outputs as a prioritized list of repurposed drugs. The results of the comparisons are shown in Table 4.

**TABLE 4:** Functional benchmarking of *in-mac* and ReBrain against existing drug-repurposing platforms for brain-related applications through comparison of four features: specificity for brain cancer, use of profile signatures for drug repurposing, repurposing based on reference drugs, and availability of a prioritized drug list. Both applications demonstrated full functionality across all categories.

Platform	Brain cancer (specific selectivity)	Profile-signature-based search capability	Repurposing based on existing drugs	Availability of a drug prioritization list
*In-mac* + ReBrain	Yes	Yes	Yes	Yes
Drug-repurposing hub (Broad Institute)	No (general, multidisease)	No	No	Partial (drug annotations without disease-specific sorting)
DisGeNET	No (disease agnostic)	No	No	No
Hetionet/Neo4j drug repurposing	No (generic network-based)	No	No	No
Open target platform	Partial (includes cancer but not selective to brain-specific repurposing)	No	No	Partial (target-centric but not drug-centric)

### 3.2 ReBrain: a network-based database

**FIGURE 13:**
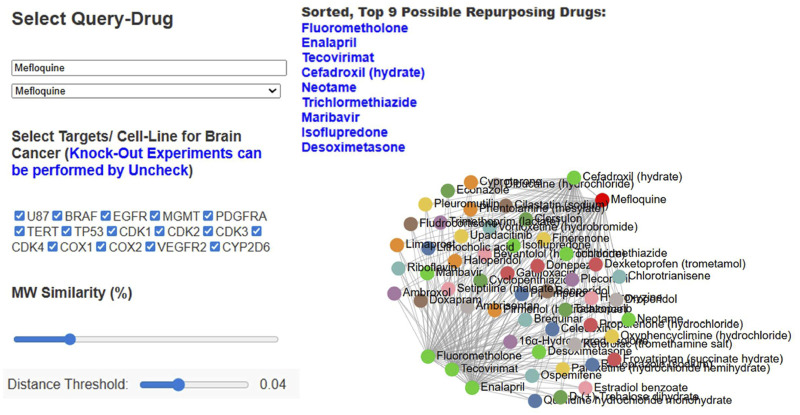
Front-end view of ReBrain, which supports text-based and drop-down menu search functions for repurposed drugs based on reference drugs. Profile component selection can be customized using checkboxes, and unchecking a profile box reflects the knockout feature of ReBrain. The network can be controlled with sliders for molecular weight and internode distance. The outputs can be visualized as a network graph or as a prioritized list of drugs referenced by the query drug.

The profiles developed for FDA-approved drug molecules were used to develop the network-based database ReBrain in this work. This database was established to support large-scale querying, visualization, and comparative analyses of repurposing candidates across brain-cancer-related molecular landscapes. This resource integrates profiling data, network relationships, and literature annotations to provide a comprehensive platform for ongoing drug-repurposing research; here, the reference drugs can be selected using a drop-down menu or a text-search option. The assay components can be chosen from preselected checkboxes, but the user is allowed to customize the selections using the checkboxes. The platform also facilitates finetuning to tailor the analysis to particular mechanistic hypotheses or therapeutic focuses in two ways: experimentation with the drug network via knock-in and knockout functionalities; and tuning the network through adjustable filters based on molecular weight and similarity distance thresholds. All processing steps were initially completed using the default settings, and the dynamic impacts of each activity were observed in the browser. The results were obtained as a network graph and as a sorted list of the top nine drug molecules. Each node in the network was connected to a relevant drug node based on its relative distance, defined on the basis of the corresponding profiles. Euclidean distances computed from these weights enabled the establishment of a multidimensional drug-repurposing network. The drug nodes were paired and linked based on matching profile intensities. Information about the drug-linked targets can be visualized by hovering over the nodes or their text (Figure 13).

### 3.3 Identification of repurposed drugs

For each reference query drug, the system generates a network and a prioritized list of possible repurposed candidates ranked by similarity in their molecular profiles. These prioritized drugs are significant because they can modulate shared oncogenic pathways implicated in brain cancers. However, before repurposing such prioritized drugs or molecules, they must be reevaluated in combination with their corresponding reference drugs. Drug–drug profile regression analysis reveals the weighted relationships between drug pairs, capturing similarities in their predicted effects on the targeted molecular components. This step is crucial because it quantifies the probability of modulation of a predicted pathway in a manner similar to that of the reference drug. Ultimately, these validations reinforce confidence in the computational predictions and highlight promising avenues for experimental and clinical exploration.

### 3.4 Repurposing of FDA-approved drugs for brain cancer

**FIGURE 14:**
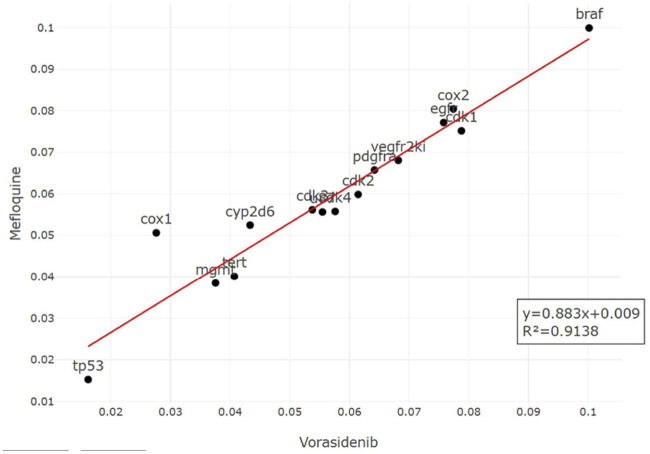
Regression plot of the top-ranked repurposed drug (mefloquine profile on y-axis) vs. its corresponding reference drug (vorasidenib profile on x-axis). The profiles were matched with an *R* ^2^ of 0.9138. Suitable biological interpretations and mechanistic insights were also found to prove the potency of mefloquine.

Belzutifan, carmustine, eflornithine, lomustine, temozolomide, and mefloquine are well-known small molecules with anti-cancer activity against brain cancers (resource: https://www.cancer.gov/about-cancer/treatment/drugs/brain). A drug-repurposing study was performed using these known FDA-approved molecules as references. For each reference drug, the highest-priority molecule was used to explain the process. The resulting repurposed drugs are summarized in Table 5. Additionally, each reference vs. repurposed drug pair was evaluated through regression analysis to quantify the profile similarities. The first reference drug processed thus was belzutifan, which was repurposed with cefaclor for an *R* ^2^ value of 0.04; this suggested low repurposing potential, as significant repurposing is considered only for *R* ^*2*^ >0.6. Another molecule, clofibric acid, was repurposed with carmustine and showed a high *R* ^2^ value of 0.90 and favored significant potential. Similarly, another low-potency repurposing was observed for eflornithine in reference to glyceryl 1-monooctanoate (*R* ^2^ = 0.17). Lomustine was repurposed with armillarisin A with an *R* ^2^ of 0.34 but a weight value of 0.97, suggesting expected repurposing except for COX-2 deviation. Neryl acetate was found to have low repurposing value in reference to temozolomide (*R* ^2^ = 0.22). Finally, vorasidenib citrate showed high confidence for drug repurposing with mefloquine, achieving an *R* ^2^ of 0.91 and a weight of 0.88 (Figure 14), which reinforced its significant potential. Based on these observations, three repurposed drugs are suggested as priority candidates, namely, mefloquine (reference: vorasidenib citrate), clofibric acid (reference: carmustine), and armillarisin A (reference: lomustine).

**TABLE 5:** Drug-repurposing outputs for six known drugs against brain cancer (belzutifan, carmustine, eflornithine, lomustine, temozolomide, and vorasidenib). These repurposed molecules were assessed via regression evaluation, which showed high plausibility for one molecule and low plausibility for two molecules (GitHub: *Supplementary_Data_Inmac_Outputs.zip* and *Supplementary_Data_RegressionPlots_Table_5.zip*).

S. no.	Reference drug	Possible repurposed drug	Functional repurposed similarity (regression coefficient R^2^ between the reference vs. repurposed drug profiles) and remarks
1	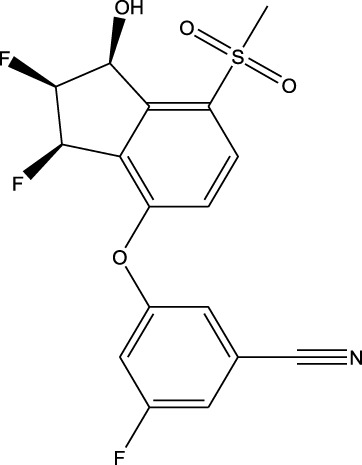 Belzutifan	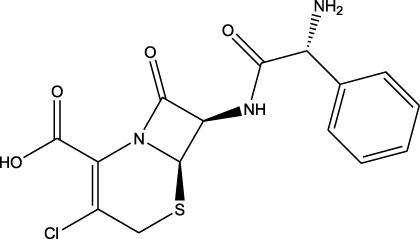 Cefaclor	*R* ^2^ = 0.0453 Non-significant profile similarity found
2	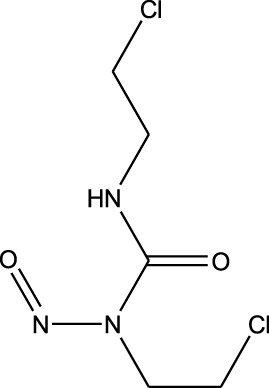 Carmustine (alkylating agent)	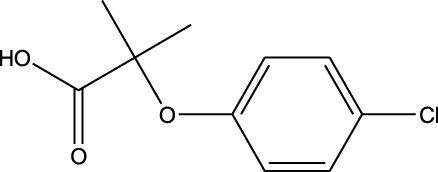 Clofibric acid (PPAR alpha agonist)	*R* ^2^ = 0.9005 Significant profile similarity found
3	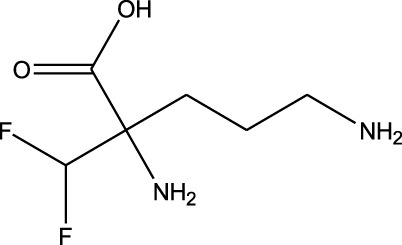 Eflornithine hydrochloride	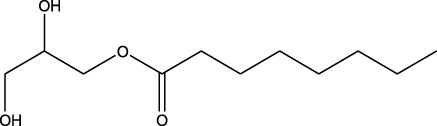 Glyceryl 1-monooctanoate	*R* ^2^ = 0.1689 Non-significant profile similarity found
4	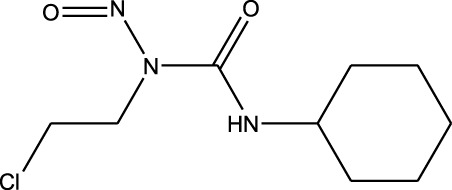 Lomustine (alkylating agent)	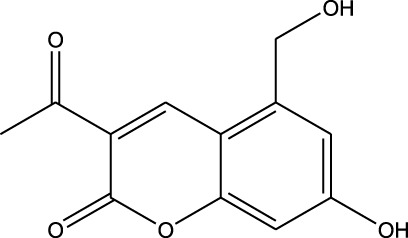 Armillarisin A (IL-4 up, IL-1β down)	*R* ^2^ = 0.3457 Non-significant profile similarity noted because of COX-2 being an outlier. A significant similarity is observed without COX-2 in the profile
5	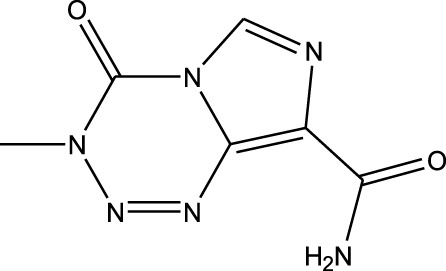 Temozolomide	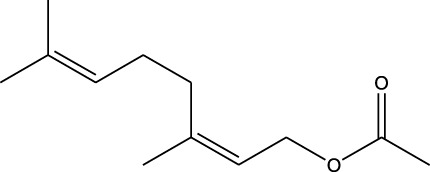 Neryl acetate	*R* ^2^ = 0.2185 Non-significant profile similarity found
6	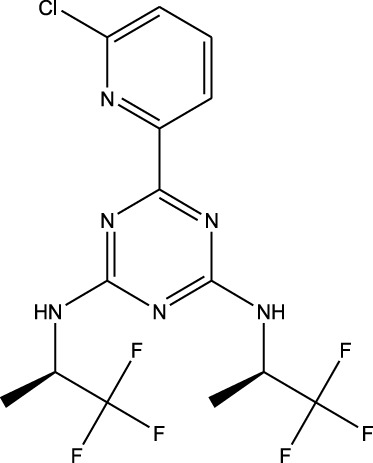 Vorasidenib citrate (anticancer activity)	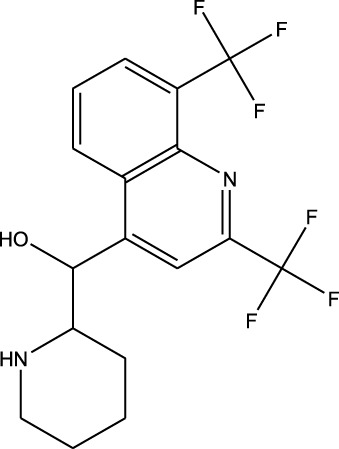 Mefloquine (antimalarial)	*R* ^2^ = 0.9138 PMID: 35417045 Significant profile similarity found

### 3.5 Molecular interactions of the final repurposed drug

**FIGURE 15:**
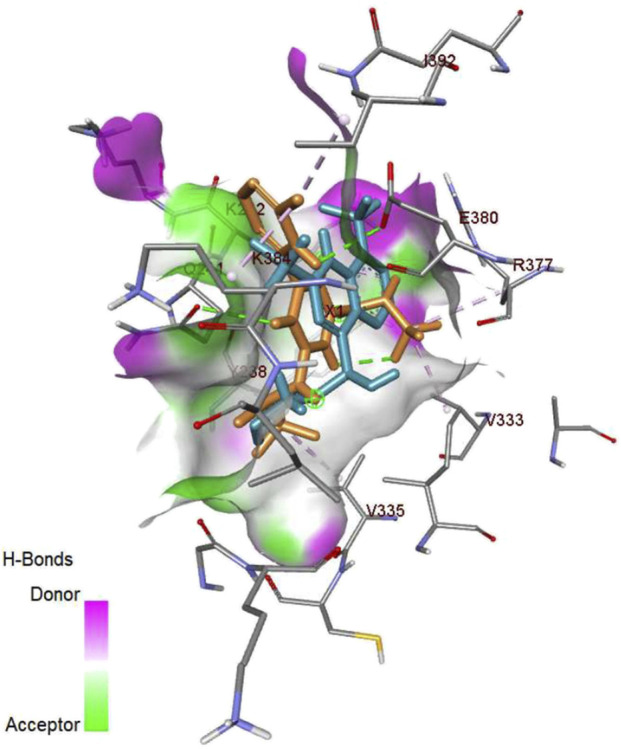
Three-dimensional representation of the molecular interactions of vorasidenib citrate and mefloquine with isocitrate dehydrogenase 1/2 (IDH1/2). Since suitable biological interpretations and mechanistic insights were found to prove the potency of mefloquine, its interaction possibilities were compared with those of the reference drug.

Herein, the top-priority repurposed drug (vorasidenib citrate) was assessed through a molecular interaction study in comparison to its reference drug, mefloquine. Since mefloquine is known to target IDH1/2 while vorasidenib citrate is originally recognized for its antimalarial activity, it was necessary to evaluate the molecular interactions of vorasidenib citrate with IDH1/2. Thus, vorasidenib citrate showed a binding affinity of −8.5 kcal/mol compared to mefloquine, which exhibited −8.4 kcal/mol within the same binding pocket (Figure 15; Supplementary Figure S1). Mefloquine (repurposed) performed better than vorasidenib (anti-cancer) in terms of crossing the BBB (Supplementary Figure S2) (*GitHub: Figures.zip*).

## 4 Discussion

The present work integrates drug profiling with network-based analysis to explore repurposing opportunities for FDA-approved small molecules for treating brain cancer. Accordingly, a drug-repurposing study was conducted in two steps by referencing known molecules targeted at brain cancers against FDA-approved drugs. A profile generator *in-mac* was defined in the first step, while a multidimensional landscape was generated based on FDA-approved drug molecules in the second step. The landscape was implemented in a database called ReBrain, where a drug similarity network was used to identify drug repurposing potential. Sufficient literature is available on pathway-specific drug repurposing efforts in the recent past (Macak et al., 2025; Jia et al., 2025; Liu and Luo, 2024; Sinha et al., 2023). Drug repurposing is also evident with FDA- and European Medicines Agency (EMA)-approved molecules (Ma et al., 2025; Huang et al., 2025). The involvement of pathway dysregulation in drug repurposing is a known process (Wang et al., 2025; Zheng et al., 2025). In the case of brain cancer, there is available evidence on repurposing neurological drugs for brain cancer (Sarker and Newland, 2025). Therefore, in the present study, the pathway components were considered for drug repurposing. Here, the pathway components form a theoretical representation of the biological system defined by SAR-based molecular profiles, and these profiles were generated using the *in-mac* application. The utility of *in-mac* can be observed in terms of the authenticity of the quantitative SAR models for predicting molecular activities. *In-mac* generates 1,000 SAR models for each query molecule and uses only these prediction models, which enables an accuracy of >80%, provided the query molecule lies within the applicability domain of each SAR model. For SAR model development, the Monte–Carlo method was adopted to capture the natural behaviors for prediction. Therefore, there may be slight deviations in the predicted values because of the randomized process. However, beyond these deviations, the application is highly accurate for population data prediction. *In-mac* creates bioassay models for activity predictions, where these bioassay models contain patterns regarding the links between structures and their activities. Fifteen specific assay components were chosen to quantify the molecules using approximately all possible pharmacodynamic and pharmacokinetic methods. This represents significant diversity coverage in defining therapeutic concerns for brain cancer. The combination of these model patterns is used to create a profile indicating the functional behaviors of the molecules in a system. These profile components are separable at the elemental level, so this method can be used for profile matching. Profile-based drug–drug functional similarity is the direct representation of drug repurposing. Here, the proposed workflow is strong as it considers global properties for decision-making, while limitations may arise with the extent of data availability in public databases. Regression entails vector elements for establishing the relationships between vectors and is therefore significant for evaluating the profile match. Profile similarities may be negative or positive; therefore, there was a need to consider the squared differences between the molecules to calculate neutral values for the similarity matrix. Since Euclidean distance computation involves this neutralization process, it was used in this study for network creation. Theoretically, the repurposing workflow used here is significant for making predictions. The present workflow can be considered as a theoretical representation of a biological system, but the results from ReBrain were proven to be authentic since they were found to have common pathways of the repurposed drugs.

The *R* ^2^ values for cefaclor vs. belzutifan (*R* ^2^ = 0.04), glyceryl 1-monooctanoate vs. eflornithine (*R* ^2^ = 0.17), and neryl acetate vs. temozolomide (*R* ^2^ = 0.22) prove that the corresponding molecules are not functionally similar at the systemic level based on the 15 profile components. The genotypic–phenotypic relationships were found to be significant when *R* ^2^ was >0.6, as in the cases of clofibric acid vs. carmustine (*R* ^2^ = 0.90) and vorasidenib citrate vs. mefloquine (*R* ^2^ = 0.91, weight = 0.88). In the case of armillarisin A vs. lomustine (*R* ^2^ = 0.34, weight = 0.97), a high weight was observed with a low *R* ^2^ value that indicated slight deviations at the anti-inflammatory activity level but good alignment in the functionalities of the other pathway components.

In terms of the biological interpretation and mechanistic insight of the prioritized repurposed drugs, the following observations are noted. (i) Mefloquine shows high potential for treating brain cancer based on various *in vitro* and *in vivo* studies, including those investigating cytotoxicity and inhibition of angiogenesis. Mefloquine is a known antimalarial molecule that targets lysosomal integrity; it induces oxidative and endoplasmic reticulum stress and can also trigger apoptosis and autophagy inhibition in tumor cells. It shows good BBB penetration and can sensitize tumor cells via inhibition of drug efflux pumps. Mefloquine is reported to act by interfering with lysosomal functions and autophagy flux; it has been shown to activate different stress pathways (JNK, ERK, and AMPK) and inhibit PI3K/Akt/mTOR signaling in several tumor models. In glioblastomas, it was shown to inhibit angiogenesis by targeting glioblastoma microvascular endothelial cells and impairing tumor vasculature (Wan et al., 2021). (ii) Clofibric acid shows low potential for direct brain cancer therapy; it acts as a PPARα agonist and is involved in fatty-acid-β oxidation, peroxisome proliferation, and xenobiotic metabolism. In this manner, it influences tumor cell lipid metabolism and oxidative stress. It also engages in PPARα signaling (Wang et al., 2010). (iii) Armillarisin A is not a plausible treatment for brain cancer at present; it is a natural product (from fungal sources) and shows antimicrobial and anti-inflammatory activities. It is known to be involved in general pathways, such as oxidative stress response, inflammatory signaling, and possibly microbial defense. No citations were found relating it to brain cancer.

The main limitations of the proposed methods here are that they have bound profile constraints for defining biological systems and lack sufficient experimental data for authentic empirical modeling of individual system components. These limitations should be addressed over time through the availability of diverse experimental datasets and the discovery of novel drug targets.

The proposed applications are suitable for observing all possible permutations and combinations of therapeutic molecules for the repurposing of not only existing but also future drugs. Furthermore, new profile components may need to be added to enhance the accuracy of deep searching of repurposed drugs. In the future, additional assay components can also be defined and included in the applications to enhance their accuracy. Although we have used machine-learning processes in this work, other options for pathway selection and phenotype predictions may be added in the future. Moreover, clinical data and adverse effect profiles can also be integrated to refine the repurposed drugs. Researchers can also supplement relevant data in terms of community-level inputs to enhance the utility of the database.

## 5 Conclusion

The present work entails rational drug repurposing by incorporating the molecular heterogeneity of brain cancers into two new computational frameworks, namely, the *in-mac* tool and the ReBrain database. Here, dysregulated pathway components were utilized to develop the molecular profiles of FDA-approved drugs using *in-mac*. Then, 2,809 FDA-approved small molecules with molecular weights ≤500 Da were profiled to create a multidimensional network-based database. The proposed profile-network-based method showed 70%–95% accuracy for drug repurposing across different disease categories related to the brain. By analyzing known drug molecules against brain cancers, we identified three repurposed drug molecules as high-priority candidates: mefloquine, clofibric acid, and armillarisin A. Of these, mefloquine was found to meet all the criteria established in this study. These findings demonstrate that system-level network analyses support drug repurposing and synergistic opportunities for the treatment of different types of brain cancers. Furthermore, additional profile components can be identified to ensure the authenticity of the profiles in drug repurposing and the synergistic utilization of the identified drugs.

## Data Availability

The original contributions presented in the study are included in the article/Supplementary Material, and any further inquiries may be directed to the corresponding authors.
