# Supplementary material for: A network-driven computational framework for identifying FDA-approved drug repurposing across heterogeneous brain cancers
Source: Front Mol Biosci. 2026 Feb 17;13:1768081. doi: 10.3389/fmolb.2026.1768081 (PMC12953378; doi:10.3389/fmolb.2026.1768081)
Supplement: Supplementary file 2 [file DataSheet6.pdf]

Supplementary Figures

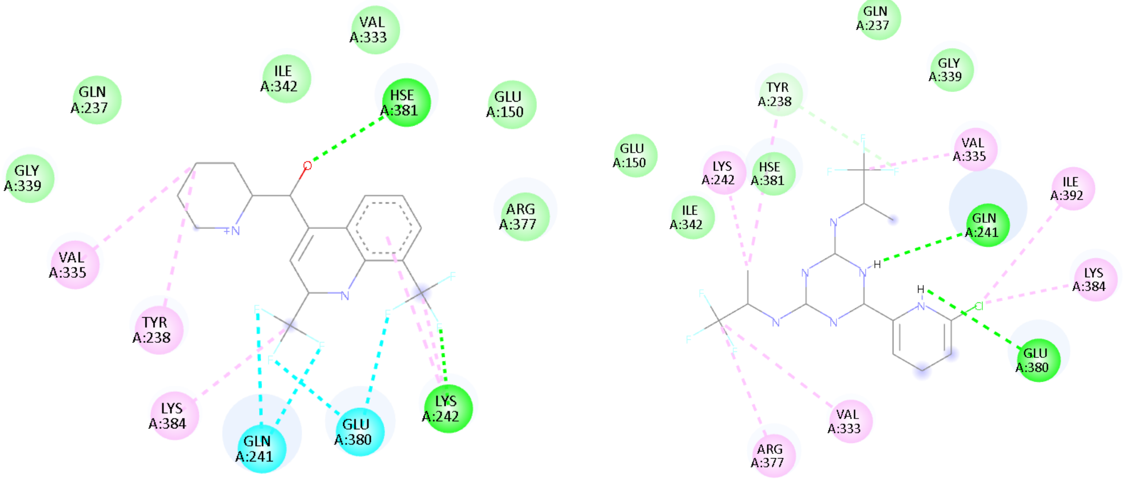

Supplementary FIGURE 1. 2D representation of molecular interaction Vorasidenib Citrate & Mefloquine with IDH1/2

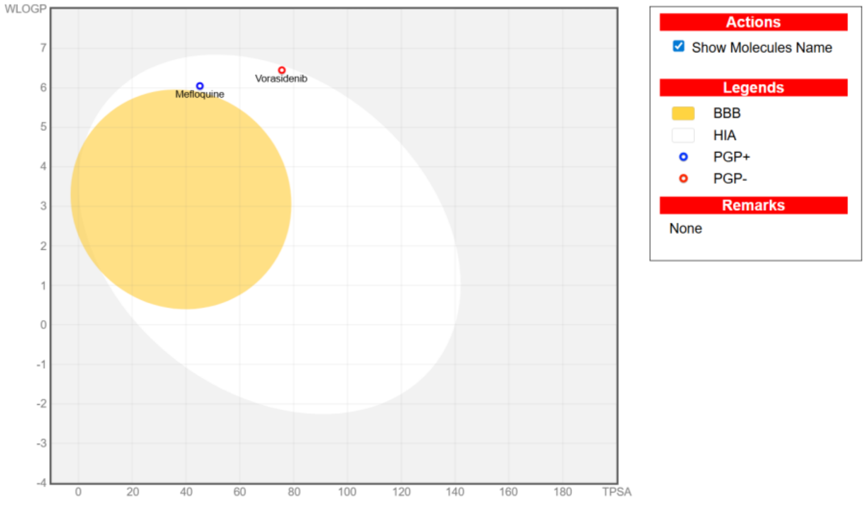

Supplementary FIGURE 2. Mefloquine (repurposed) performed better than Vorasidenib (anticancer) for crossing the BBB
