# Supplementary material for: A network-driven computational framework for identifying FDA-approved drug repurposing across heterogeneous brain cancers
Source: Front Mol Biosci. 2026 Feb 17;13:1768081. doi: 10.3389/fmolb.2026.1768081 (PMC12953378; doi:10.3389/fmolb.2026.1768081)
Supplement: Supplementary file 3 [file DataSheet1.zip › Supplementary_Data_Inmac_Outputs/ArmillarisinA_Escorwin_BioAssay_Report.pdf]

Escorwin Innovations Pvt. Ltd.

CIN: U21000UP2025PTC221194, GSTIN: 09AAICE8098B1ZC

In-macs Computational Bioassay Report

Query SMILES: CC(=O)c2cc1c(CO)cc(O)cc1oc2=O
Assay Environment: Target/CellLine, R2avg, SARactivity, SARstd, inmacActivity, inmacResolution

Assay Environment: CDK1 (G1/M),0.89709,7.91697,0.87403,0.09036,5.02516
Assay Environment: CDK2 (G1/S),0.90053,6.61643,0.63180,0.07014,4.37167
Assay Environment: CDK3 (G0/G1),Infinity,7.21147,0.86919,0.06511,5.12858
Assay Environment: CDK4 (G1),0.89545,6.87734,0.66330,0.06162,4.90543
Assay Environment: VEGFR2,0.88833,5.32513,0.51187,0.05853,3.45197
Assay Environment: TP53,0.84430,4.83902,0.07045,0.00961,4.53141
Assay Environment: Amyloidbeta,Infinity,4.64301,0.42485,0.04900,3.07551
Assay Environment: BRAF,0.94667,6.22481,0.35450,0.04842,4.67530
Assay Environment: EGFR,0.86792,5.40951,0.85950,0.05622,3.61021
Assay Environment: MGMT,0.89603,5.97721,0.52754,0.13871,1.53811
Assay Environment: PDGFRA,0.88332,6.33211,0.29127,0.02939,5.39158
Assay Environment: TERT,0.88409,4.56891,0.47291,0.03075,3.58497
Assay Environment: EGFR1975,0.94251,5.35203,0.07438,0.01293,4.93833
Assay Environment: EGFR226,0.89276,3.58400,0.95859,0.05772,1.73689
Assay Environment: COX1,0.86507,5.51385,0.76875,0.09268,2.54785
Assay Environment: COX2,0.88257,5.59674,0.61707,0.09257,2.63430
Assay Environment: Inha,0.84636,5.44095,0.47411,0.04190,4.10003
Assay Environment: U87,0.87383,4.81543,0.40022,0.03192,3.79382
Assay Environment: Tubulin,0.88082,5.11522,0.31264,0.03748,3.91579
Assay Environment: GABA Human,0.86803,7.19832,0.53538,0.05871,5.31960
Assay Environment: GABA Rat,0.87529,6.17849,0.88129,0.08462,3.47049
Assay Environment: CYP2D6,0.87243,4.73569,0.47269,0.03732,3.54120

Authorized Signatory

Quality & Compliance, Escorwin Inno. Pvt. Ltd.

Generated on: 10/12/2025 10:12
