# Supplementary material for: A network-driven computational framework for identifying FDA-approved drug repurposing across heterogeneous brain cancers
Source: Front Mol Biosci. 2026 Feb 17;13:1768081. doi: 10.3389/fmolb.2026.1768081 (PMC12953378; doi:10.3389/fmolb.2026.1768081)
Supplement: Supplementary file 3 [file DataSheet1.zip › Supplementary_Data_Inmac_Outputs/Belzutifan_Escorwin_BioAssay_Report.pdf]

Escorwin Innovations Pvt. Ltd.

CIN: U21000UP2025PTC221194, GSTIN: 09AAICE8098B1ZC

In-macs Computational Bioassay Report

Query SMILES: CS(=O)(=O)c2ccc(Oc1cc(F)cc(C#N)c1)c3c2[C@H](O)[C@H](F)[C@@H]3F
Assay Environment: Target/CellLine, R2avg, SARactivity, SARstd, inmacActivity, inmacResolution

Assay Environment: CDK1 (G1/M),0.89372,9.03636,1.10467,0.08932,5.04335
Assay Environment: CDK2 (G1/S),0.89867,7.44295,0.74935,0.06609,4.48876
Assay Environment: CDK3 (G0/G1),Infinity,7.85638,1.01770,0.06346,5.02041
Assay Environment: CDK4 (G1),0.89406,7.58637,0.74043,0.06244,4.79532
Assay Environment: VEGFR2,0.88148,5.91967,0.64481,0.07145,2.72548
Assay Environment: TP53,0.86903,4.74515,0.14191,0.01196,4.21050
Assay Environment: Amyloidbeta,0.90060,5.26470,0.39664,0.04750,3.14124
Assay Environment: BRAF,0.87926,6.06767,0.90660,0.07538,2.69806
Assay Environment: EGFR,0.87615,5.83477,0.87301,0.06899,2.75094
Assay Environment: MGMT,0.87598,6.55019,0.68428,0.06990,3.42529
Assay Environment: PDGFRA,0.87393,6.22753,0.61819,0.05723,3.66920
Assay Environment: TERT,0.86948,4.94590,0.46785,0.03244,3.49559
Assay Environment: EGFR1975,0.91292,5.54409,0.10616,0.01229,4.99484
Assay Environment: EGFR226,0.88106,4.39311,0.74447,0.06095,1.66858
Assay Environment: COX1,0.87626,5.99144,1.16715,0.17705,-1.92311
Assay Environment: COX2,Infinity,6.63168,0.56322,0.10829,1.79280
Assay Environment: Inha,0.87761,5.41193,0.55771,0.06870,2.34068
Assay Environment: U87,0.87204,4.96426,0.52810,0.03923,3.21067
Assay Environment: Tubulin,0.88001,5.50971,0.37177,0.03509,3.94103
Assay Environment: GABA Human,0.86773,7.62089,0.77119,0.09001,3.59730
Assay Environment: GABA Rat,0.87514,6.91309,0.97539,0.12551,1.30243
Assay Environment: CYP2D6,0.87062,5.09909,0.52609,0.04684,3.00503

Authorized Signatory
Quality & Compliance, Escorwin Inno. Pvt. Ltd.

Generated on: 10/12/2025 10:00
