# Supplementary material for: A network-driven computational framework for identifying FDA-approved drug repurposing across heterogeneous brain cancers
Source: Front Mol Biosci. 2026 Feb 17;13:1768081. doi: 10.3389/fmolb.2026.1768081 (PMC12953378; doi:10.3389/fmolb.2026.1768081)
Supplement: Supplementary file 3 [file DataSheet1.zip › Supplementary_Data_Inmac_Outputs/Carmustine_Escorwin_BioAssay_Report.pdf]

In-macs Computational Bioassay Report

Query SMILES: O=NN(CCCl)C(=O)NCCCl
Assay Environment: Target/CellLine, R2avg, SARactivity, SARstd, inmacActivity, inmacResolution

Assay Environment: CDK1 (G1/M),Infinity,7.07364,0.86774,0.09329,4.95580
Assay Environment: CDK2 (G1/S),0.90393,5.94388,0.64488,0.07172,4.31510
Assay Environment: CDK3 (G0/G1),0.88165,6.54164,0.85550,0.06763,5.00574
Assay Environment: CDK4 (G1),0.89560,6.30738,0.69674,0.06139,4.91337
Assay Environment: VEGFR2,0.88292,4.75375,0.39981,0.05581,3.48645
Assay Environment: TP53,NaN,NaN,NaN,NaN,NaN
Assay Environment: Amyloidbeta,0.91432,4.13937,0.48319,0.04957,3.01374
Assay Environment: BRAF,NaN,NaN,NaN,NaN,NaN
Assay Environment: EGFR,0.84280,5.84234,1.13849,0.03892,4.95839
Assay Environment: MGMT,0.90362,4.77578,0.30053,0.14489,1.48538
Assay Environment: PDGFRA,NaN,NaN,NaN,NaN,NaN
Assay Environment: TERT,0.85998,4.40082,0.46883,0.02544,3.82316
Assay Environment: EGFR1975,0.96073,5.14954,0.03768,0.01442,4.82218
Assay Environment: EGFR226,0.89645,3.51210,0.98694,0.04847,2.41132
Assay Environment: COX1,0.85935,5.09657,0.59817,0.06541,3.61108
Assay Environment: COX2,0.88016,5.37971,0.51839,0.06078,3.99949
Assay Environment: Inha,0.85922,5.23715,0.44756,0.03706,4.39563
Assay Environment: U87,0.84887,4.70528,0.52788,0.02739,4.08321
Assay Environment: Tubulin,NaN,NaN,NaN,NaN,NaN
Assay Environment: GABA Human,0.87346,6.68185,0.21095,0.05817,5.36094
Assay Environment: GABA Rat,0.87904,5.85722,0.85184,0.06972,4.27394
Assay Environment: CYP2D6,0.88439,4.69142,0.47956,0.02793,4.05707

Authorized Signatory
Quality & Compliance, Escorwin Inno. Pvt. Ltd.

Generated on: 10/12/2025 10:04
