# Supplementary material for: A network-driven computational framework for identifying FDA-approved drug repurposing across heterogeneous brain cancers
Source: Front Mol Biosci. 2026 Feb 17;13:1768081. doi: 10.3389/fmolb.2026.1768081 (PMC12953378; doi:10.3389/fmolb.2026.1768081)
Supplement: Supplementary file 3 [file DataSheet1.zip › Supplementary_Data_Inmac_Outputs/Cefaclor_Escorwin_BioAssay_Report.pdf]

Escorwin Innovations Pvt. Ltd.
CIN: U21000UP2025PTC221194, GSTIN: 09AAICE8098B1ZC

In-macs Computational Bioassay Report

Query SMILES: N[C@@H](C(=O)N[C@@H]1C(=O)N2C(C(=O)O)=C(Cl)CS[C@H]12)c3ccccc3
Assay Environment: Target/CellLine, R2avg, SARactivity, SARstd, inmacActivity, inmacResolution

Assay Environment: CDK1 (G1/M),0.89234,9.77285,1.17808,0.07967,5.21934
Assay Environment: CDK2 (G1/S),Infinity,8.04229,0.84292,0.06016,4.60454
Assay Environment: CDK3 (G0/G1),Infinity,8.21228,1.01161,0.05582,5.02341
Assay Environment: CDK4 (G1),0.89293,8.16725,0.80319,0.05819,4.84127
Assay Environment: VEGFR2,0.88212,6.66086,0.72639,0.07531,2.35678
Assay Environment: TP53,0.87251,4.81989,0.20912,0.01404,4.01738
Assay Environment: Amyloidbeta,0.89548,5.75826,0.40397,0.04453,3.21345
Assay Environment: BRAF,0.87733,6.67062,0.81979,0.09210,1.40690
Assay Environment: EGFR,0.87211,6.41394,0.88552,0.07749,1.98488
Assay Environment: MGMT,0.88061,6.81681,0.71808,0.04694,4.13400
Assay Environment: PDGFRA,0.87051,6.71429,0.71234,0.07141,2.63308
Assay Environment: TERT,0.87460,5.26464,0.47550,0.03685,3.15836
Assay Environment: EGFR1975,0.91034,5.68476,0.09627,0.01317,4.93180
Assay Environment: EGFR226,0.88719,4.93564,0.76326,0.07081,0.88855
Assay Environment: COX1,0.88718,5.40041,0.57556,0.04979,2.55477
Assay Environment: COX2,0.87460,7.31062,0.69273,0.08535,2.43257
Assay Environment: Inha,0.87728,5.90171,0.76355,0.08629,0.96974
Assay Environment: U87,0.87576,5.22429,0.68651,0.05369,2.15566
Assay Environment: Tubulin,Infinity,5.74706,0.35242,0.03291,3.86688
Assay Environment: GABA Human,0.86198,7.79154,0.71938,0.06821,3.89294
Assay Environment: GABA Rat,0.87271,7.58580,0.92289,0.10451,1.61242
Assay Environment: CYP2D6,0.86821,5.40280,0.61256,0.05072,2.50397

Authorized Signatory
Quality & Compliance, Escorwin Inno. Pvt. Ltd.

Generated on: 10/12/2025 10:03
