# Supplementary material for: A network-driven computational framework for identifying FDA-approved drug repurposing across heterogeneous brain cancers
Source: Front Mol Biosci. 2026 Feb 17;13:1768081. doi: 10.3389/fmolb.2026.1768081 (PMC12953378; doi:10.3389/fmolb.2026.1768081)
Supplement: Supplementary file 3 [file DataSheet1.zip › Supplementary_Data_Inmac_Outputs/ClofibricAcid_Escorwin_BioAssay_Report.pdf]

Escorwin Innovations Pvt. Ltd.

CIN: U21000UP2025PTC221194, GSTIN: 09AAICE8098B1ZC

In-macs Computational Bioassay Report

Query SMILES: CC(C)(Oc1ccc(Cl)cc1)C(=O)O
Assay Environment: Target/CellLine, R2avg, SARactivity, SARstd, inmacActivity, inmacResolution

Assay Environment: CDK1 (G1/M),0.90899,5.64819,0.82083,0.09751,4.63959
Assay Environment: CDK2 (G1/S),0.91550,4.73272,0.63449,0.08031,3.90210
Assay Environment: CDK3 (G0/G1),0.88518,5.37091,0.73048,0.07484,4.59684
Assay Environment: CDK4 (G1),0.90239,5.39610,0.73527,0.06562,4.71736
Assay Environment: VEGFR2,NaN,NaN,NaN,NaN,NaN
Assay Environment: TP53,NaN,NaN,NaN,NaN,NaN
Assay Environment: Amyloidbeta,0.92656,3.20050,0.73056,0.05780,2.60264
Assay Environment: BRAF,NaN,NaN,NaN,NaN,NaN
Assay Environment: EGFR,0.98639,5.90346,0.00000,0.03194,5.57315
Assay Environment: MGMT,NaN,NaN,NaN,NaN,NaN
Assay Environment: PDGFRA,NaN,NaN,NaN,NaN,NaN
Assay Environment: TERT,0.96124,6.31366,0.00000,0.00778,6.23322
Assay Environment: EGFR1975,NaN,NaN,NaN,NaN,NaN
Assay Environment: EGFR226,NaN,NaN,NaN,NaN,NaN
Assay Environment: COX1,NaN,NaN,NaN,NaN,NaN
Assay Environment: COX2,NaN,NaN,NaN,NaN,NaN
Assay Environment: Inha,NaN,NaN,NaN,NaN,NaN
Assay Environment: U87,NaN,NaN,NaN,NaN,NaN
Assay Environment: Tubulin,NaN,NaN,NaN,NaN,NaN
Assay Environment: GABA Human,NaN,NaN,NaN,NaN,NaN
Assay Environment: GABA Rat,0.87288,5.06378,0.72136,0.06428,4.39894
Assay Environment: CYP2D6,NaN,NaN,NaN,NaN,NaN

Authorized Signatory
Quality & Compliance, Escorwin Inno. Pvt. Ltd.

Generated on: 10/12/2025 10:06
