# Supplementary material for: A network-driven computational framework for identifying FDA-approved drug repurposing across heterogeneous brain cancers
Source: Front Mol Biosci. 2026 Feb 17;13:1768081. doi: 10.3389/fmolb.2026.1768081 (PMC12953378; doi:10.3389/fmolb.2026.1768081)
Supplement: Supplementary file 3 [file DataSheet1.zip › Supplementary_Data_Inmac_Outputs/Eflornithine_Escorwin_BioAssay_Report.pdf]

Escorwin Innovations Pvt. Ltd.
CIN: U21000UP2025PTC221194, GSTIN: 09AAICE8098B1ZC

In-macs Computational Bioassay Report

Query SMILES: NCCCC(N)(C(=O)O)C(F)F
Assay Environment: Target/CellLine, R2avg, SARactivity, SARstd, inmacActivity, inmacResolution

Assay Environment: CDK1 (G1/M),0.90768,5.98771,0.83384,0.09520,4.79003
Assay Environment: CDK2 (G1/S),0.91059,5.05619,0.65414,0.07397,4.12558
Assay Environment: CDK3 (G0/G1),0.89031,5.68580,0.80191,0.07377,4.75775
Assay Environment: CDK4 (G1),Infinity,5.66133,0.68207,0.06351,4.86289
Assay Environment: VEGFR2,NaN,NaN,NaN,NaN,NaN
Assay Environment: TP53,NaN,NaN,NaN,NaN,NaN
Assay Environment: Amyloidbeta,0.92473,3.44900,0.70238,0.05565,2.74886
Assay Environment: BRAF,NaN,NaN,NaN,NaN,NaN
Assay Environment: EGFR,0.93453,5.29196,0.25267,0.03446,4.85840
Assay Environment: MGMT,NaN,NaN,NaN,NaN,NaN
Assay Environment: PDGFRA,NaN,NaN,NaN,NaN,NaN
Assay Environment: TERT,NaN,NaN,NaN,NaN,NaN
Assay Environment: EGFR1975,NaN,NaN,NaN,NaN,NaN
Assay Environment: EGFR226,0.89969,2.25699,0.24975,0.05931,1.51080
Assay Environment: COX1,0.88103,4.86258,0.24888,0.05786,4.13464
Assay Environment: COX2,NaN,NaN,NaN,NaN,NaN
Assay Environment: Inha,NaN,NaN,NaN,NaN,NaN
Assay Environment: U87,0.88944,4.60863,0.34768,0.02090,4.34574
Assay Environment: Tubulin,NaN,NaN,NaN,NaN,NaN
Assay Environment: GABA Human,NaN,NaN,NaN,NaN,NaN
Assay Environment: GABA Rat,0.88407,5.64758,1.02882,0.06142,4.87489
Assay Environment: CYP2D6,NaN,NaN,NaN,NaN,NaN

Authorized Signatory
Quality & Compliance, Escorwin Inno. Pvt. Ltd.

Generated on: 10/12/2025 10:08
