# Supplementary material for: A network-driven computational framework for identifying FDA-approved drug repurposing across heterogeneous brain cancers
Source: Front Mol Biosci. 2026 Feb 17;13:1768081. doi: 10.3389/fmolb.2026.1768081 (PMC12953378; doi:10.3389/fmolb.2026.1768081)
Supplement: Supplementary file 3 [file DataSheet1.zip › Supplementary_Data_Inmac_Outputs/Glyceryl_1-monooctanoate_Escorwin_BioAssay_Report.pdf]

Escorwin Innovations Pvt. Ltd.

CIN: U21000UP2025PTC221194, GSTIN: 09AAICE8098B1ZC

In-macs Computational Bioassay Report

Query SMILES: CCCCCCCC(=O)OCC(O)CO
Assay Environment: Target/CellLine, R2avg, SARactivity, SARstd, inmacActivity, inmacResolution

Assay Environment: CDK1 (G1/M),0.89910,7.35374,0.85702,0.09209,4.98023
Assay Environment: CDK2 (G1/S),0.90157,6.17535,0.60396,0.07085,4.34921
Assay Environment: CDK3 (G0/G1),0.88441,6.82463,0.83692,0.06733,5.08921
Assay Environment: CDK4 (G1),0.89838,6.51365,0.66710,0.06167,4.92415
Assay Environment: VEGFR2,0.89437,4.91579,0.49668,0.05755,3.43255
Assay Environment: TP53,NaN,NaN,NaN,NaN,NaN
Assay Environment: Amyloidbeta,0.91246,4.30338,0.44646,0.05092,2.99092
Assay Environment: BRAF,0.80326,6.69504,0.88477,0.01959,6.19004
Assay Environment: EGFR,0.88686,5.28267,1.02187,0.04968,4.00233
Assay Environment: MGMT,0.89745,5.19832,0.37014,0.13808,1.63943
Assay Environment: PDGFRA,NaN,NaN,NaN,NaN,NaN
Assay Environment: TERT,0.86453,4.67936,0.59200,0.02352,4.07324
Assay Environment: EGFR1975,0.96360,5.18809,0.03332,0.01449,4.81467
Assay Environment: EGFR226,0.88170,3.56001,0.90127,0.04673,2.35559
Assay Environment: COX1,0.87215,5.17958,0.70122,0.07142,3.33885
Assay Environment: COX2,0.87189,5.62765,0.46739,0.06224,4.02345
Assay Environment: Inha,0.85387,5.47296,0.44339,0.03703,4.51850
Assay Environment: U87,0.87945,4.75225,0.48569,0.02975,3.98552
Assay Environment: Tubulin,NaN,NaN,NaN,NaN,NaN
Assay Environment: GABA Human,0.87568,7.02551,0.40934,0.04694,5.81563
Assay Environment: GABA Rat,0.87801,5.82455,0.84019,0.07633,3.85731
Assay Environment: CYP2D6,0.85918,4.63675,0.41694,0.03318,3.78167

Authorized Signatory

Quality & Compliance, Escorwin Inno. Pvt. Ltd.

Generated on: 10/12/2025 10:09
