# Supplementary material for: A network-driven computational framework for identifying FDA-approved drug repurposing across heterogeneous brain cancers
Source: Front Mol Biosci. 2026 Feb 17;13:1768081. doi: 10.3389/fmolb.2026.1768081 (PMC12953378; doi:10.3389/fmolb.2026.1768081)
Supplement: Supplementary file 3 [file DataSheet1.zip › Supplementary_Data_Inmac_Outputs/Lomustine_Escorwin_BioAssay_Report.pdf]

In-macs Computational Bioassay Report

Query SMILES: O=NN(CCCl)C(=O)NC1CCCCC1
Assay Environment: Target/CellLine, R2avg, SARactivity, SARstd, inmacActivity, inmacResolution

Assay Environment: CDK1 (G1/M),Infinity,8.67789,1.00490,0.08897,5.09093
Assay Environment: CDK2 (G1/S),Infinity,7.18585,0.69299,0.06699,4.48586
Assay Environment: CDK3 (G0/G1),Infinity,7.66101,0.92123,0.06271,5.13326
Assay Environment: CDK4 (G1),0.89259,7.36573,0.69591,0.06178,4.87437
Assay Environment: VEGFR2,0.88452,5.75954,0.59060,0.06502,3.13761
Assay Environment: TP53,0.87049,4.70175,0.15096,0.01147,4.23932
Assay Environment: Amyloidbeta,Infinity,5.07271,0.39613,0.04785,3.14348
Assay Environment: BRAF,0.91023,5.98747,0.85626,0.06080,3.53574
Assay Environment: EGFR,0.87662,5.76053,0.89486,0.06627,3.08834
Assay Environment: MGMT,0.87325,6.39910,0.58505,0.08528,2.96025
Assay Environment: PDGFRA,0.88723,5.95232,0.69192,0.05964,3.54741
Assay Environment: TERT,0.87636,4.76440,0.48173,0.03251,3.45345
Assay Environment: EGFR1975,0.92814,5.49360,0.10630,0.01218,5.00244
Assay Environment: EGFR226,0.87986,4.24418,0.76387,0.05891,1.86864
Assay Environment: COX1,0.87256,5.75223,0.93115,0.11881,0.96118
Assay Environment: COX2,Infinity,6.21786,0.54624,0.09800,2.26808
Assay Environment: Inha,0.87535,5.34294,0.57360,0.05974,2.93406
Assay Environment: U87,0.87363,4.95290,0.43801,0.03823,3.41147
Assay Environment: Tubulin,Infinity,5.42511,0.35701,0.03552,3.99377
Assay Environment: GABA Human,0.86596,7.47518,0.62579,0.07052,4.63148
Assay Environment: GABA Rat,0.87846,6.55598,0.98047,0.10920,2.15267
Assay Environment: CYP2D6,0.86330,4.98529,0.49947,0.04204,3.29024

Authorized Signatory
Quality & Compliance, Escorwin Inno. Pvt. Ltd.

Generated on: 10/12/2025 10:11
