# Supplementary material for: A network-driven computational framework for identifying FDA-approved drug repurposing across heterogeneous brain cancers
Source: Front Mol Biosci. 2026 Feb 17;13:1768081. doi: 10.3389/fmolb.2026.1768081 (PMC12953378; doi:10.3389/fmolb.2026.1768081)
Supplement: Supplementary file 3 [file DataSheet1.zip › Supplementary_Data_Inmac_Outputs/Mefloquine_Escorwin_BioAssay_Report.pdf]

In-macs Computational Bioassay Report

Query SMILES: OC(c1cc(C(F)(F)F)nc2c(C(F)(F)F)cccc12)C3CCCCN3
Assay Environment: Target/CellLine, R2avg, SARactivity, SARstd, inmacActivity, inmacResolution

Assay Environment: CDK1 (G1/M),Infinity,7.75878,0.86525,0.09391,4.98408
Assay Environment: CDK2 (G1/S),0.90016,6.45422,0.61947,0.06855,4.42837
Assay Environment: CDK3 (G0/G1),Infinity,7.04769,0.89676,0.06527,5.12082
Assay Environment: CDK4 (G1),0.89437,6.72596,0.65699,0.06261,4.87552
Assay Environment: VEGFR2,0.89068,5.10180,0.52219,0.05925,3.35079
Assay Environment: TP53,0.83979,4.81863,0.08016,0.00930,4.54385
Assay Environment: Amyloidbeta,0.90689,4.53028,0.43645,0.05108,3.02070
Assay Environment: BRAF,0.93285,6.09062,0.36367,0.03921,4.93170
Assay Environment: EGFR,0.85807,5.18832,0.76039,0.06034,3.40495
Assay Environment: MGMT,0.89598,5.64133,0.47329,0.14086,1.47837
Assay Environment: PDGFRA,0.90535,6.57195,0.09072,0.02916,5.71015
Assay Environment: TERT,0.90043,4.49379,0.43726,0.02847,3.65239
Assay Environment: EGFR1975,0.96319,5.24337,0.03371,0.01435,4.81922
Assay Environment: EGFR226,0.87376,3.78928,0.82339,0.04991,2.31438
Assay Environment: COX1,0.87406,5.36649,0.70364,0.08479,2.86077
Assay Environment: COX2,0.88300,5.50550,0.64816,0.08283,3.05767
Assay Environment: Inha,0.85474,5.36775,0.49687,0.04224,4.11938
Assay Environment: U87,0.86663,4.74720,0.40012,0.03010,3.85773
Assay Environment: Tubulin,0.86760,5.07629,0.35921,0.03975,3.90147
Assay Environment: GABA Human,0.87887,6.98753,0.55201,0.05888,5.24758
Assay Environment: GABA Rat,0.87855,6.01636,0.88628,0.08265,3.57391
Assay Environment: CYP2D6,0.87264,4.75618,0.42569,0.03402,3.75087

Authorized Signatory
Quality & Compliance, Escorwin Inno. Pvt. Ltd.

Generated on: 10/12/2025 10:21
