# Supplementary material for: A network-driven computational framework for identifying FDA-approved drug repurposing across heterogeneous brain cancers
Source: Front Mol Biosci. 2026 Feb 17;13:1768081. doi: 10.3389/fmolb.2026.1768081 (PMC12953378; doi:10.3389/fmolb.2026.1768081)
Supplement: Supplementary file 3 [file DataSheet1.zip › Supplementary_Data_Inmac_Outputs/Neryl_acetate_Escorwin_BioAssay_Report.pdf]

In-macs Computational Bioassay Report

Query SMILES: C/C(C)=C\CC/C(C)=C\COC(C)=O
Assay Environment: Target/CellLine, R2avg, SARactivity, SARstd, inmacActivity, inmacResolution

Assay Environment: CDK1 (G1/M),0.89891,8.05768,0.88670,0.09099,5.02957
Assay Environment: CDK2 (G1/S),0.90024,6.72130,0.63989,0.06904,4.42355
Assay Environment: CDK3 (G0/G1),Infinity,7.23835,0.87716,0.06393,5.11156
Assay Environment: CDK4 (G1),0.89463,6.96689,0.66779,0.06249,4.88729
Assay Environment: VEGFR2,0.88729,5.36624,0.54615,0.06085,3.34124
Assay Environment: TP53,0.86596,4.85568,0.07170,0.00909,4.55309
Assay Environment: Amyloidbeta,0.90531,4.72141,0.41557,0.04879,3.09762
Assay Environment: BRAF,0.86191,6.23580,0.42295,0.04786,4.64319
Assay Environment: EGFR,0.88593,5.35835,0.79588,0.05880,3.40159
Assay Environment: MGMT,0.89405,6.14279,0.57487,0.13674,1.59213
Assay Environment: PDGFRA,0.90039,6.37216,0.21815,0.03176,5.31508
Assay Environment: TERT,0.89291,4.50876,0.44333,0.03188,3.44779
Assay Environment: EGFR1975,0.93770,5.32989,0.07892,0.01375,4.87247
Assay Environment: EGFR226,0.88142,3.67885,0.89832,0.05786,1.75332
Assay Environment: COX1,0.85707,5.58216,0.77727,0.09544,2.40588
Assay Environment: COX2,0.88251,5.64747,0.61203,0.09418,2.51312
Assay Environment: Inha,0.86954,5.39968,0.46732,0.04519,3.89592
Assay Environment: U87,0.87204,4.81429,0.38206,0.03272,3.72533
Assay Environment: Tubulin,0.87249,5.19121,0.31057,0.03507,4.02426
Assay Environment: GABA Human,0.86544,7.31658,0.50457,0.06102,5.28577
Assay Environment: GABA Rat,0.88140,6.26023,0.83313,0.08606,3.39631
Assay Environment: CYP2D6,0.86885,4.81405,0.50919,0.03768,3.55993

Authorized Signatory
Quality & Compliance, Escorwin Inno. Pvt. Ltd.

Generated on: 10/12/2025 10:17
