# Supplementary material for: A network-driven computational framework for identifying FDA-approved drug repurposing across heterogeneous brain cancers
Source: Front Mol Biosci. 2026 Feb 17;13:1768081. doi: 10.3389/fmolb.2026.1768081 (PMC12953378; doi:10.3389/fmolb.2026.1768081)
Supplement: Supplementary file 3 [file DataSheet1.zip › Supplementary_Data_Inmac_Outputs/Temozolomide_Escorwin_BioAssay_Report.pdf]

Escorwin Innovations Pvt. Ltd.
CIN: U21000UP2025PTC221194, GSTIN: 09AAICE8098B1ZC

In-macs Computational Bioassay Report

Query SMILES: Cn2nnc1c(C(N)=O)ncn1c2=O
Assay Environment: Target/CellLine, R2avg, SARactivity, SARstd, inmacActivity, inmacResolution

Assay Environment: CDK1 (G1/M),Infinity,6.89938,0.86633,0.09518,4.86535
Assay Environment: CDK2 (G1/S),0.90535,5.83666,0.63841,0.07224,4.29137
Assay Environment: CDK3 (G0/G1),0.88870,6.47096,0.87339,0.06896,4.99572
Assay Environment: CDK4 (G1),0.89654,6.23271,0.72868,0.06270,4.89157
Assay Environment: VEGFR2,0.89418,4.70687,0.39842,0.05505,3.52939
Assay Environment: TP53,NaN,NaN,NaN,NaN,NaN
Assay Environment: Amyloidbeta,0.91174,4.08060,0.49660,0.05050,3.00029
Assay Environment: BRAF,NaN,NaN,NaN,NaN,NaN
Assay Environment: EGFR,0.89340,5.21409,0.56025,0.04722,4.20394
Assay Environment: MGMT,0.90754,4.38103,0.24183,0.14587,1.26063
Assay Environment: PDGFRA,NaN,NaN,NaN,NaN,NaN
Assay Environment: TERT,0.86278,6.37685,0.00000,0.00718,6.22334
Assay Environment: EGFR1975,0.95940,5.12669,0.03613,0.01452,4.81619
Assay Environment: EGFR226,0.90863,2.86827,0.66437,0.05567,1.67751
Assay Environment: COX1,0.86321,5.04545,0.61056,0.06183,3.72276
Assay Environment: COX2,0.88044,5.26860,0.55654,0.05826,4.02245
Assay Environment: Inha,0.83682,5.29810,0.45588,0.03445,4.56121
Assay Environment: U87,0.87848,4.64786,0.48581,0.02871,4.03371
Assay Environment: Tubulin,NaN,NaN,NaN,NaN,NaN
Assay Environment: GABA Human,NaN,NaN,NaN,NaN,NaN
Assay Environment: GABA Rat,0.86930,5.82160,0.84739,0.06630,4.40333
Assay Environment: CYP2D6,0.89251,4.61862,0.49801,0.02701,4.04082

Authorized Signatory
Quality & Compliance, Escorwin Inno. Pvt. Ltd.

Generated on: 10/12/2025 10:15
