# Supplementary material for: A network-driven computational framework for identifying FDA-approved drug repurposing across heterogeneous brain cancers
Source: Front Mol Biosci. 2026 Feb 17;13:1768081. doi: 10.3389/fmolb.2026.1768081 (PMC12953378; doi:10.3389/fmolb.2026.1768081)
Supplement: Supplementary file 3 [file DataSheet1.zip › Supplementary_Data_Inmac_Outputs/Vorasidenib_Escorwin_BioAssay_Report.pdf]

Escorwin Innovations Pvt. Ltd.
CIN: U21000UP2025PTC221194, GSTIN: 09AAICE8098B1ZC

In-macs Computational Bioassay Report

Query SMILES: C[C@@H](Nc2nc(N[C@H](C)C(F)(F)F)nc(c1cccc(Cl)n1)n2)C(F)(F)F
Assay Environment: Target/CellLine, R2avg, SARactivity, SARstd, inmacActivity, inmacResolution

Assay Environment: CDK1 (G1/M),0.89609,7.83231,0.86411,0.09218,5.01541
Assay Environment: CDK2 (G1/S),0.90199,6.51947,0.64312,0.06989,4.38390
Assay Environment: CDK3 (G0/G1),0.87808,7.10697,0.90221,0.06447,5.13691
Assay Environment: CDK4 (G1),0.89567,6.75986,0.65138,0.06071,4.90456
Assay Environment: VEGFR2,0.88494,5.21962,0.51404,0.05904,3.41553
Assay Environment: TP53,0.85900,4.83228,0.07169,0.00876,4.56451
Assay Environment: Amyloidbeta,0.90891,4.56584,0.42131,0.04943,3.05530
Assay Environment: BRAF,0.87202,6.25904,0.75410,0.03980,5.04290
Assay Environment: EGFR,0.87881,5.39504,0.85938,0.05168,3.81567
Assay Environment: MGMT,0.89651,5.78240,0.50584,0.14087,1.47760
Assay Environment: PDGFRA,0.87677,6.61551,0.16292,0.02713,5.78649
Assay Environment: TERT,0.89127,4.39282,0.34407,0.03282,3.38992
Assay Environment: EGFR1975,0.96521,5.25773,0.03309,0.01485,4.80386
Assay Environment: EGFR226,0.89231,3.53589,0.86799,0.05496,1.85656
Assay Environment: COX1,0.86581,5.38426,0.72045,0.08559,2.76882
Assay Environment: COX2,0.88257,5.57401,0.60595,0.08430,2.99792
Assay Environment: Inha,0.85992,5.33264,0.45900,0.04236,4.03810
Assay Environment: U87,0.85833,4.75863,0.39724,0.03062,3.82306
Assay Environment: Tubulin,0.86954,5.07269,0.32142,0.03629,3.96372
Assay Environment: GABA Human,0.87570,7.11297,0.51270,0.06007,5.27748
Assay Environment: GABA Rat,0.88095,6.04648,0.84644,0.08409,3.47673
Assay Environment: CYP2D6,0.85961,4.78447,0.53215,0.03661,3.66570

Authorized Signatory
Quality & Compliance, Escorwin Inno. Pvt. Ltd.

Generated on: 10/12/2025 10:19
