# Supplementary figures and images for: A network-driven computational framework for identifying FDA-approved drug repurposing across heterogeneous brain cancers
Source: Front Mol Biosci. 2026 Feb 17;13:1768081. doi: 10.3389/fmolb.2026.1768081 (PMC12953378; doi:10.3389/fmolb.2026.1768081)

Cefaclor vs Belzutifan

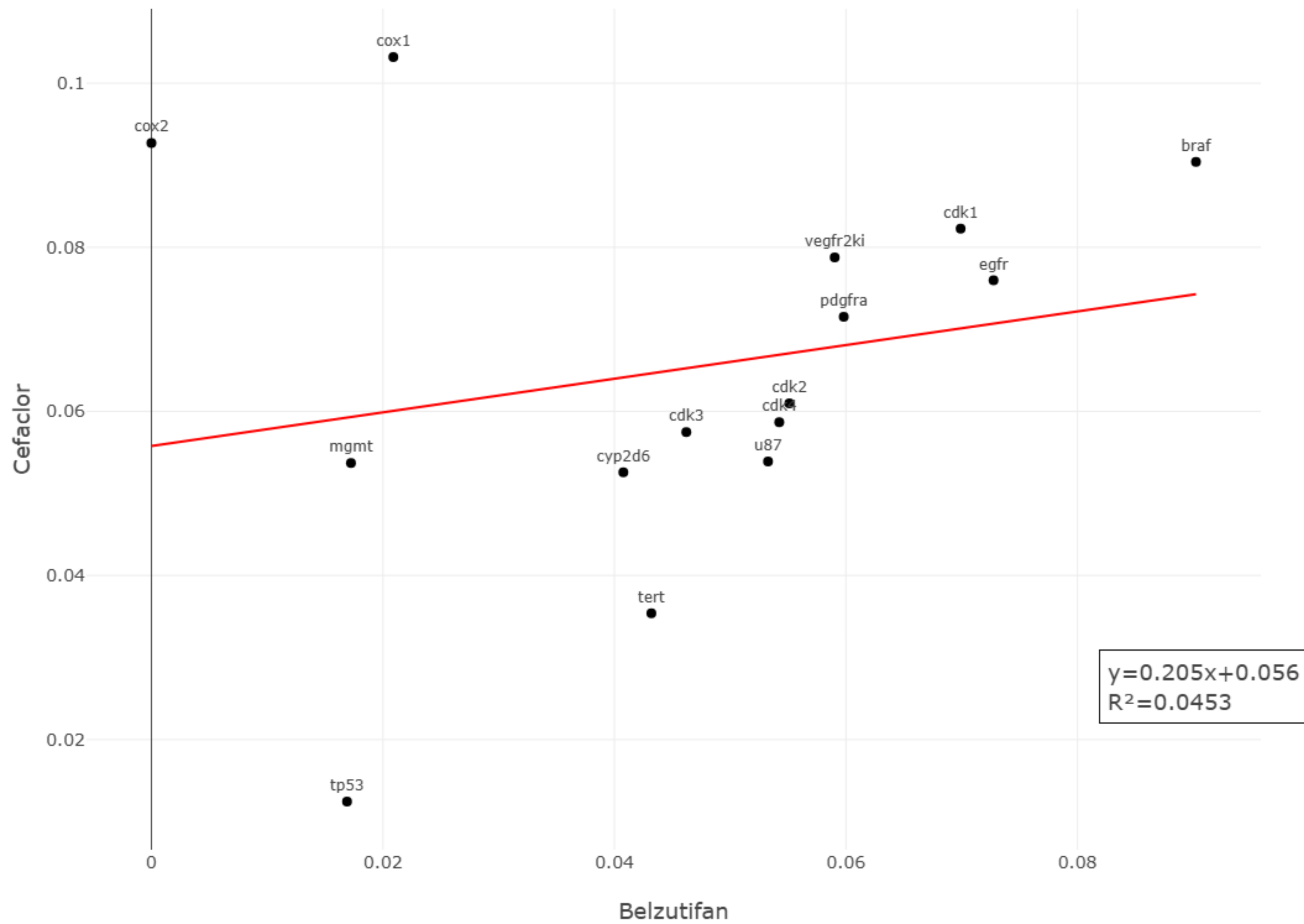

Supplement: Supplementary file 4 [file DataSheet2.zip › Supplementary_Data_RegressionPlots_Table_5/Belzutifan_Cefaclor.pdf]

Clofibric Acid vs Carmustine

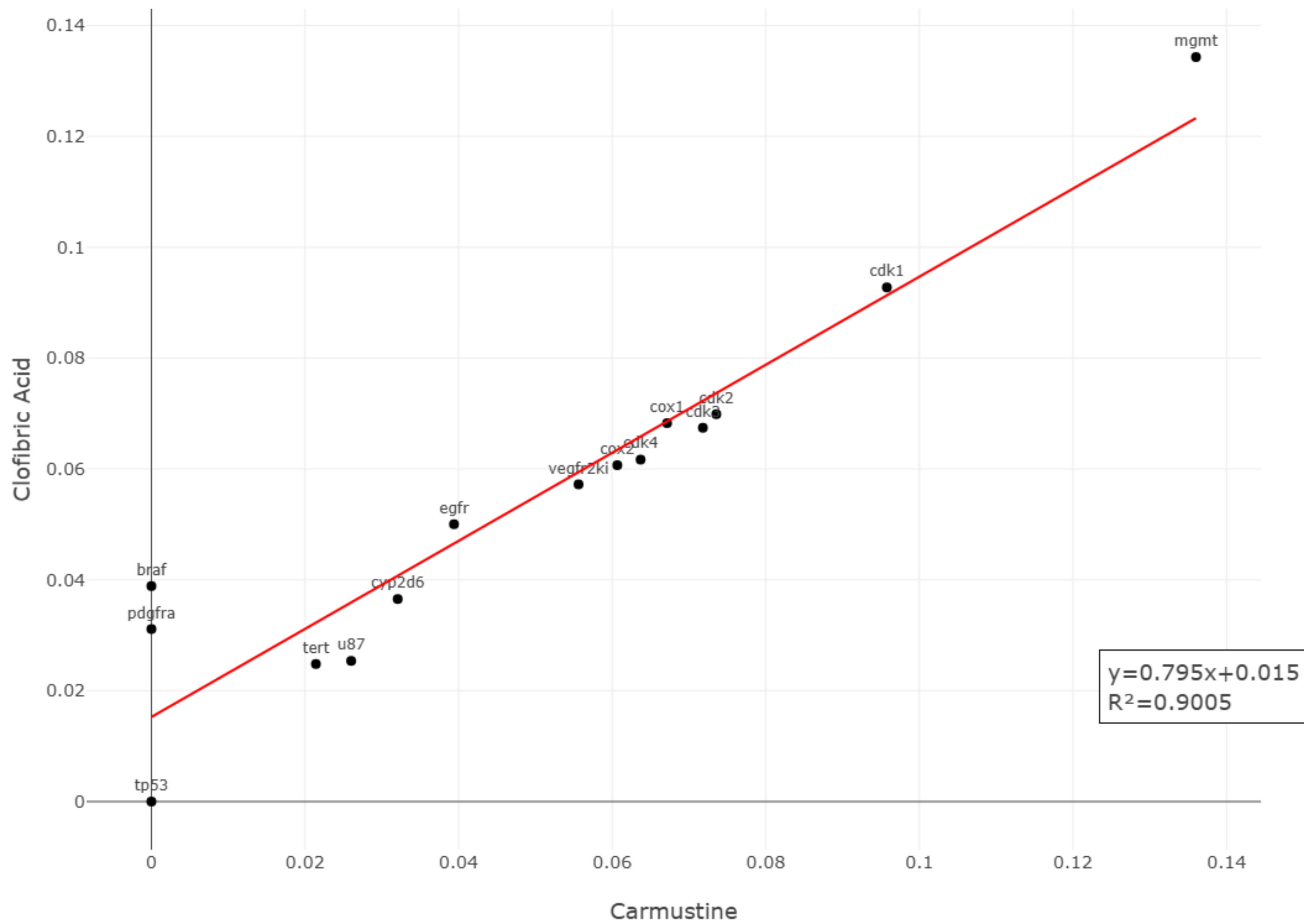

Supplement: Supplementary file 4 [file DataSheet2.zip › Supplementary_Data_RegressionPlots_Table_5/Carmustine_ClofibricAcid.pdf]

Glycerol 1-monooctanoate vs Eflornithine

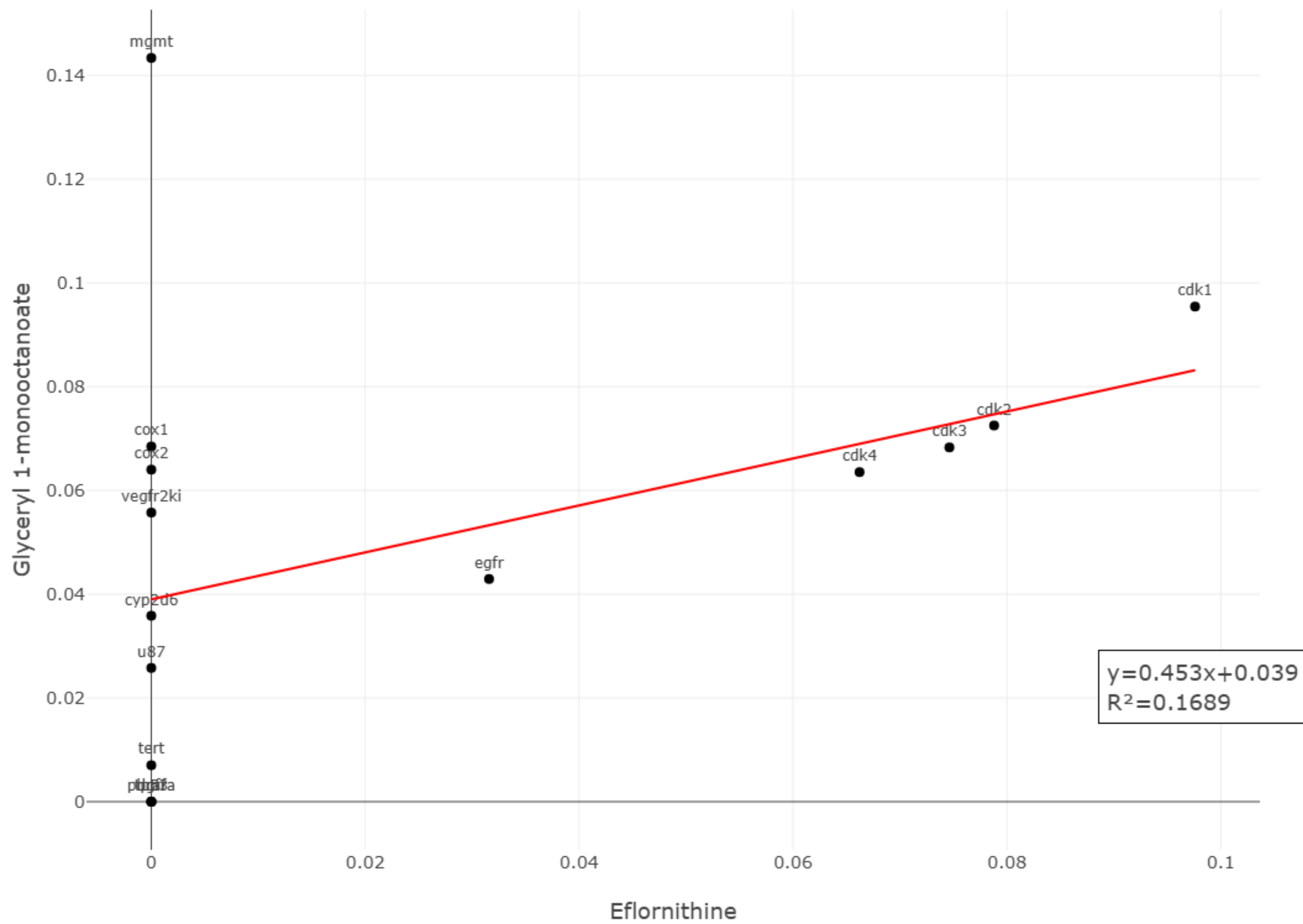

Supplement: Supplementary file 4 [file DataSheet2.zip › Supplementary_Data_RegressionPlots_Table_5/Eflornithine_Glyceryl1-monooctanoate.pdf]

Armilarisin A vs Lomustine

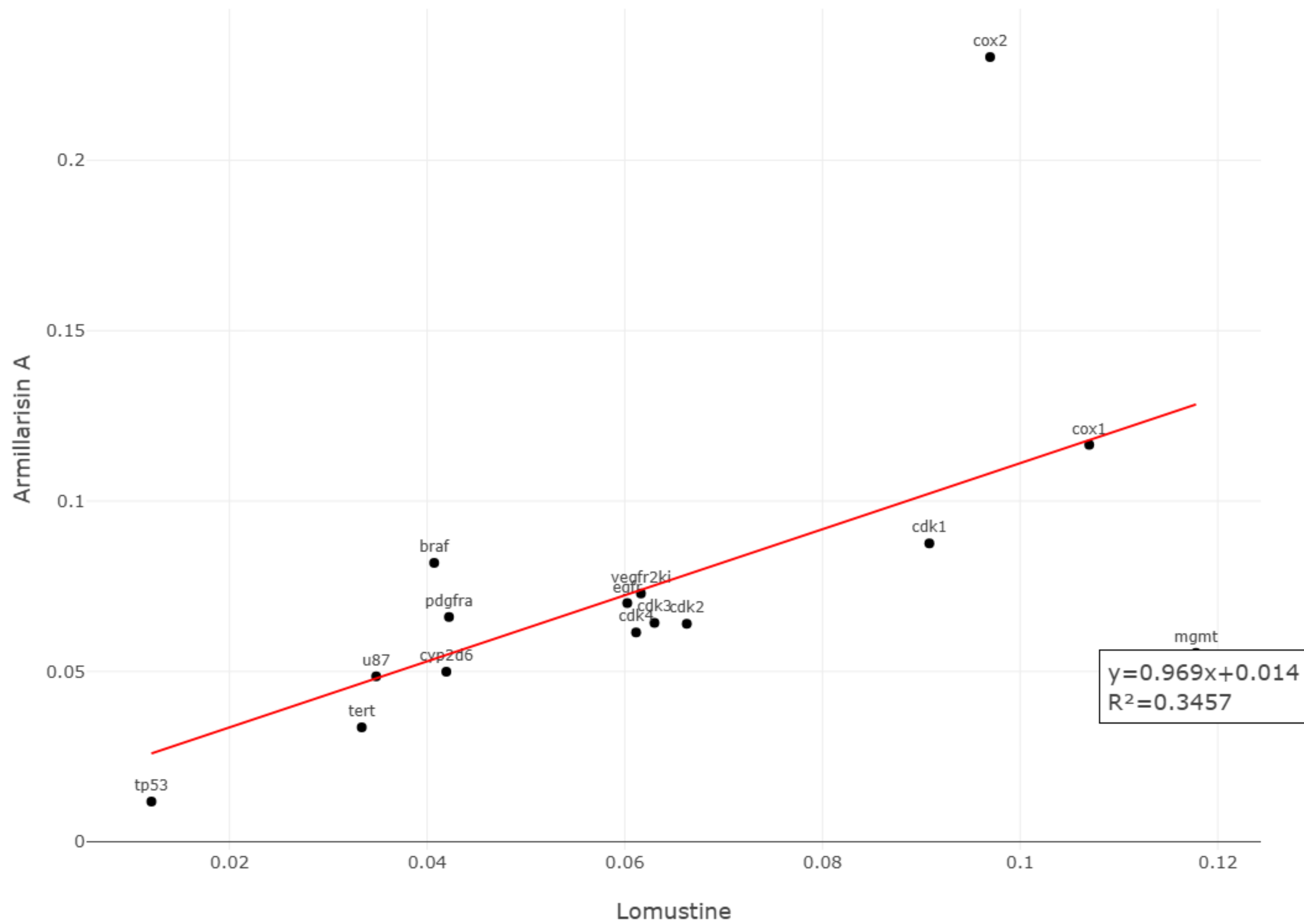

Supplement: Supplementary file 4 [file DataSheet2.zip › Supplementary_Data_RegressionPlots_Table_5/Lomustine_ArmillarisinA.pdf]

Neryl acetate vs Temozolomide

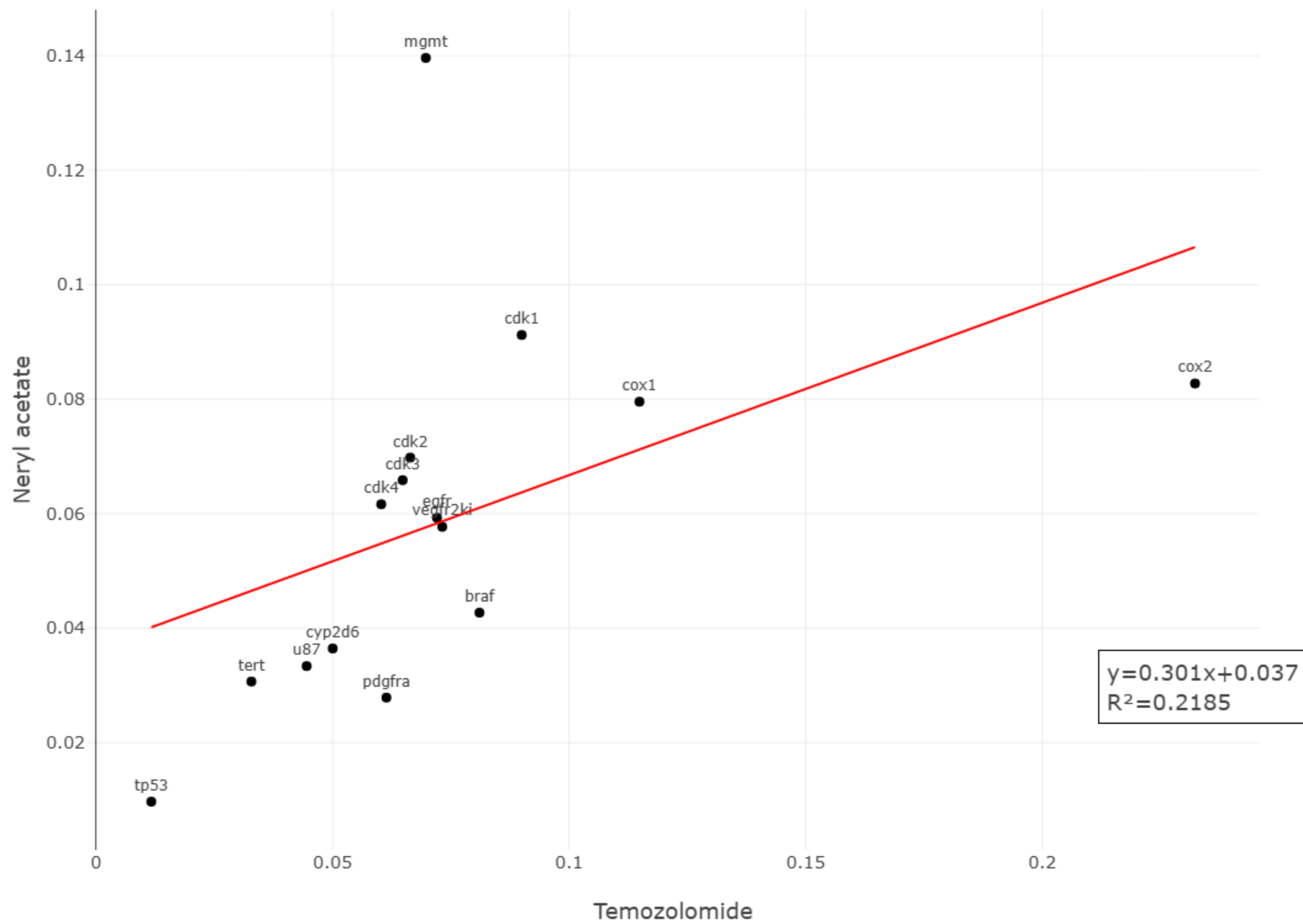

Supplement: Supplementary file 4 [file DataSheet2.zip › Supplementary_Data_RegressionPlots_Table_5/Temozolomide_Nerylacetate.pdf]

Mefloquine vs Vorasidenib

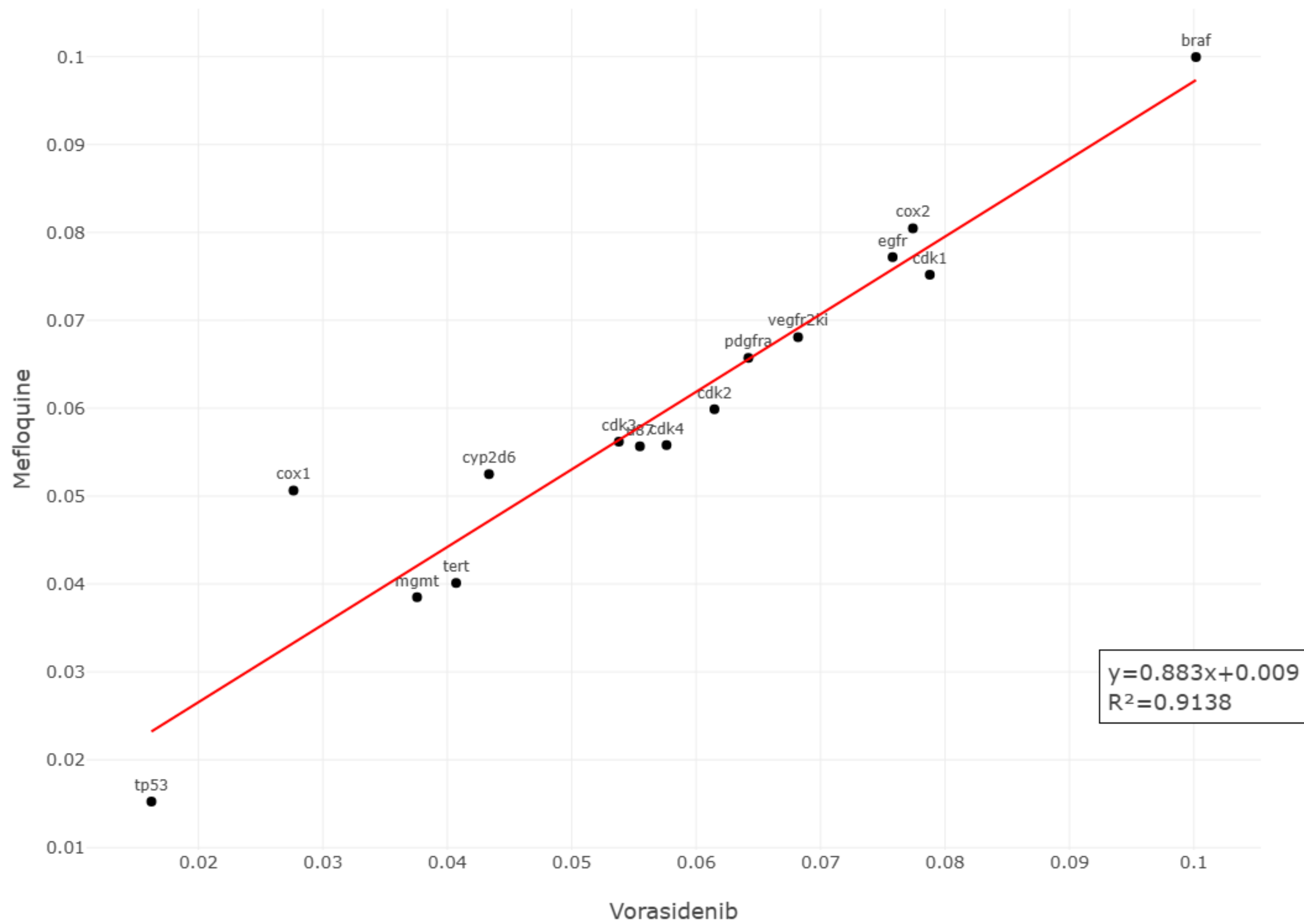

Supplement: Supplementary file 4 [file DataSheet2.zip › Supplementary_Data_RegressionPlots_Table_5/Vorasidenib_Mefloquine.pdf]

Mefloquine vs Vorasidenib

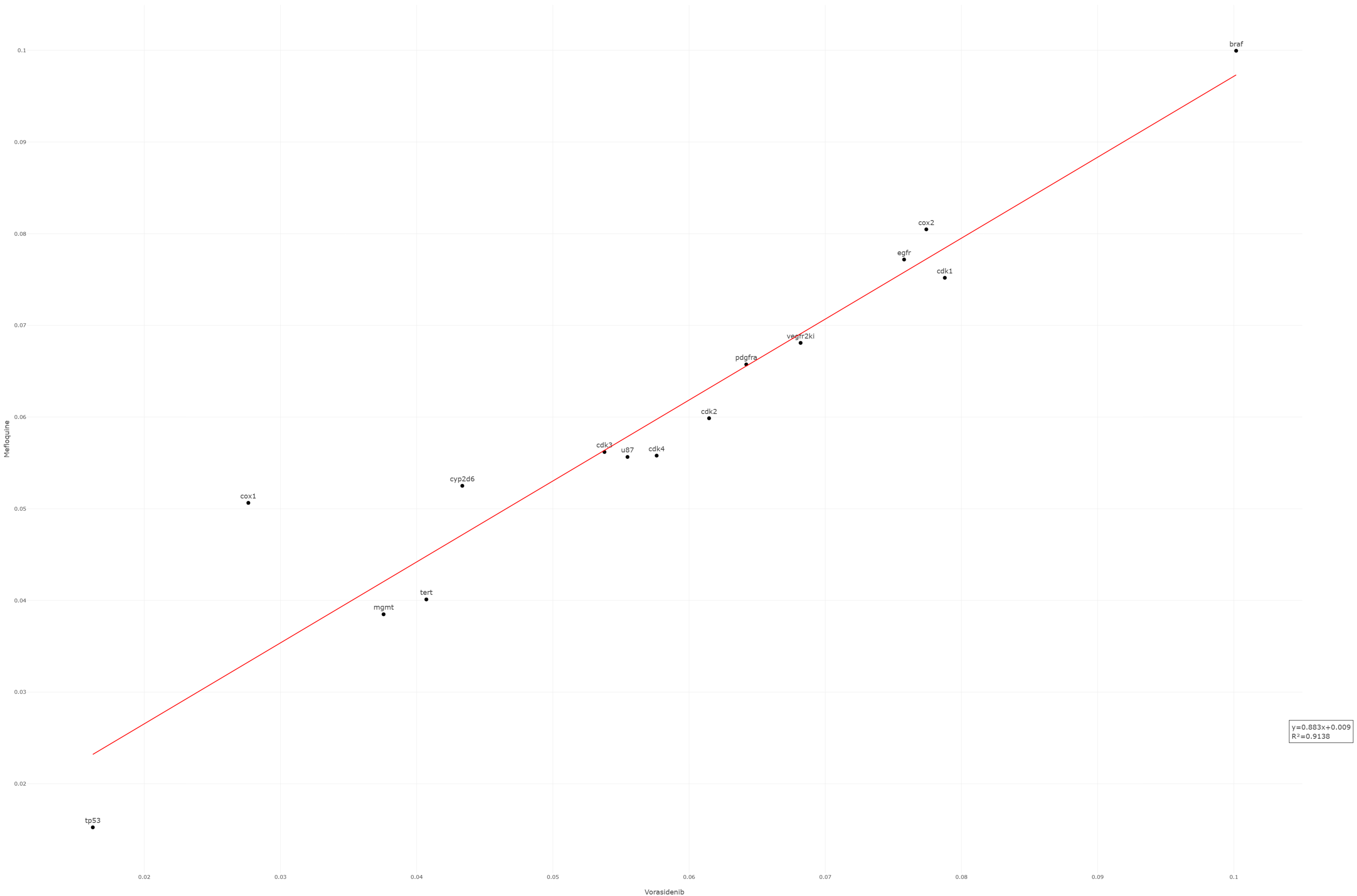

Supplement: Supplementary file 4 [file DataSheet2.zip › Supplementary_Data_RegressionPlots_Table_5/Vorasidenib_Mefloquine_Revised.pdf]

ROC Curve - ANN (AUC = 1.0000)

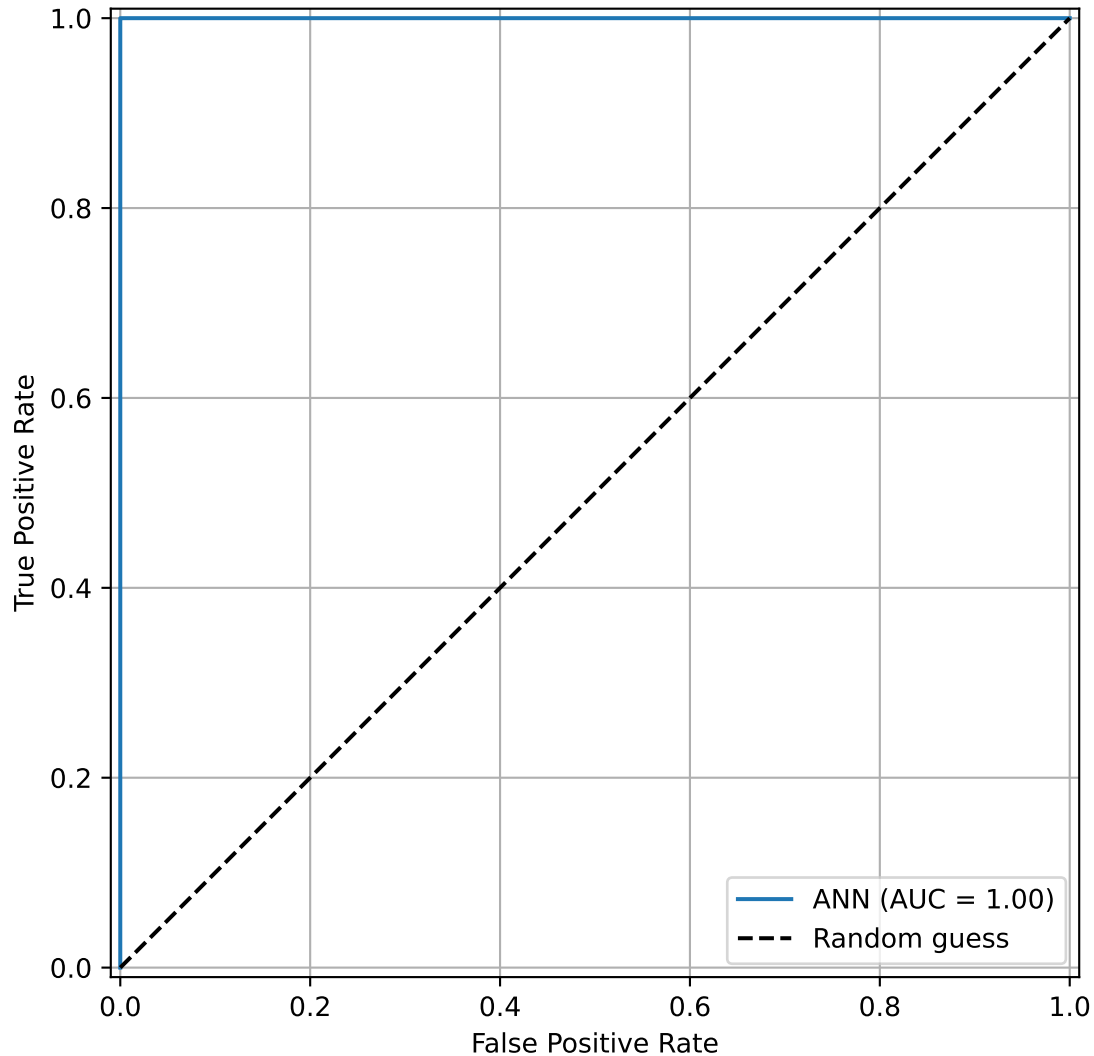

Supplement: Supplementary file 5 [file DataSheet5.zip › Supplementary_Data_Evaluation_Validation/Cancer_NonCancer/ann_roc.pdf]

ROC Curve - Decision Tree (AUC = 1.0000)

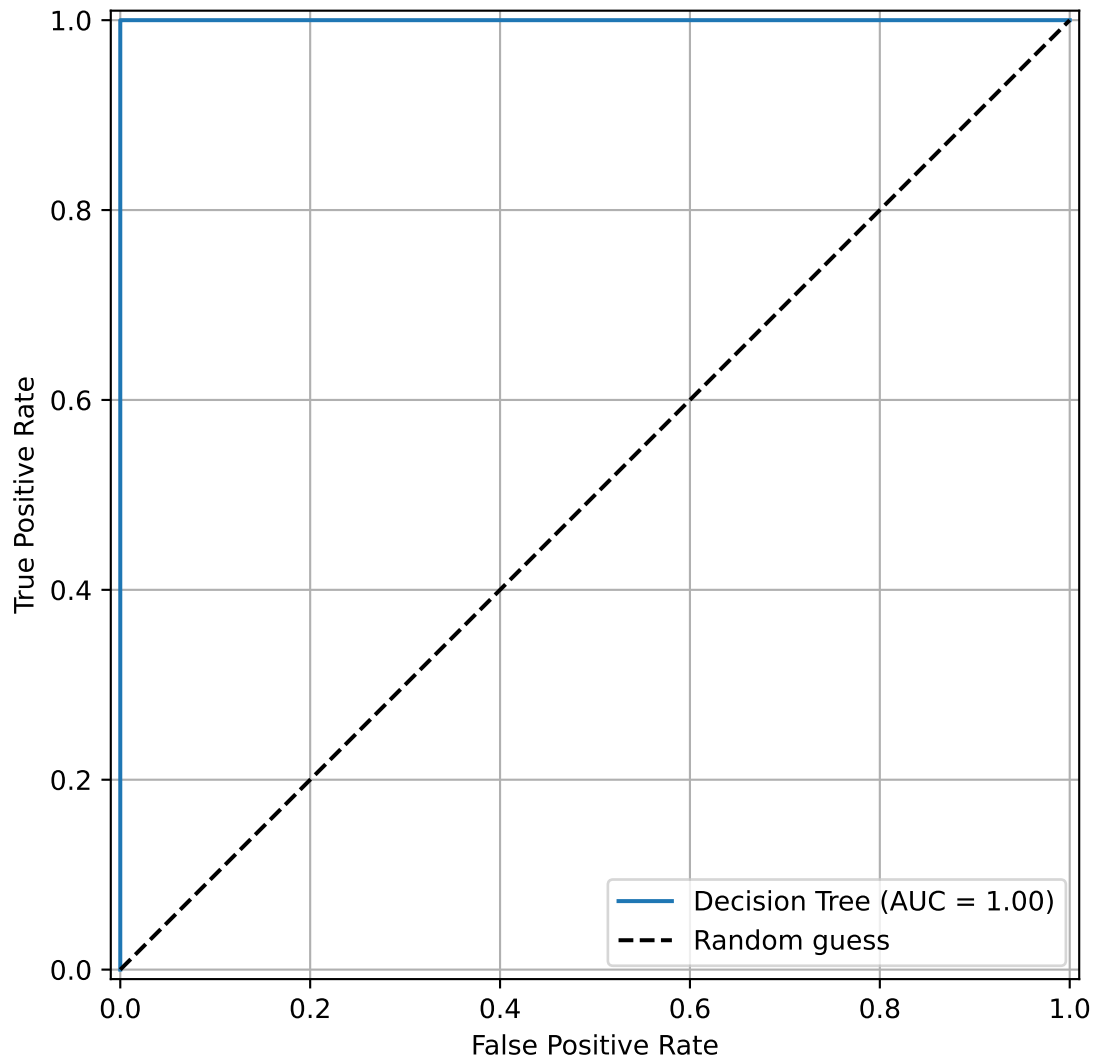

Supplement: Supplementary file 5 [file DataSheet5.zip › Supplementary_Data_Evaluation_Validation/Cancer_NonCancer/decision_tree_roc.pdf]

ROC Curve - Logistic Regression (AUC = 0.9999)

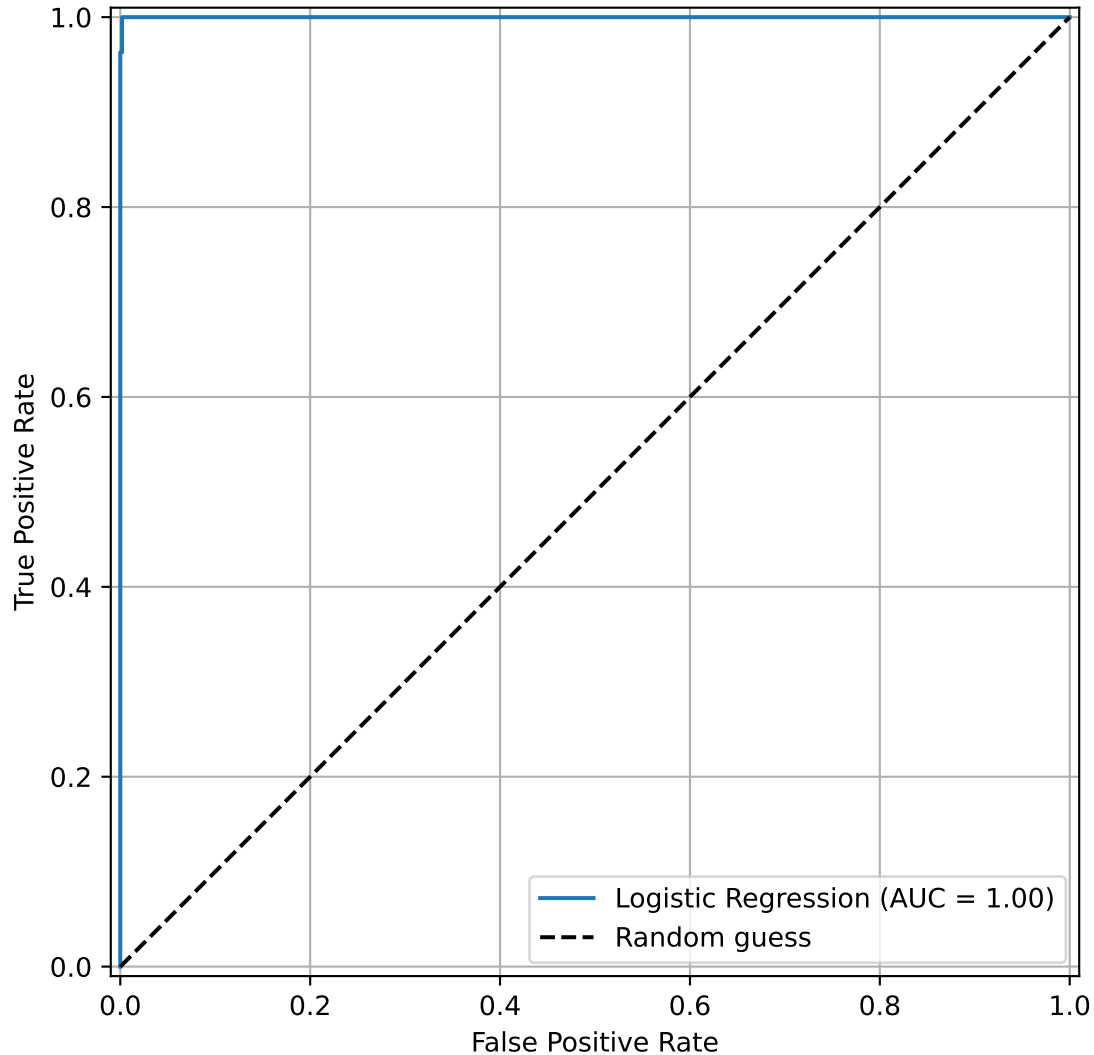

Supplement: Supplementary file 5 [file DataSheet5.zip › Supplementary_Data_Evaluation_Validation/Cancer_NonCancer/logistic_regression_roc.pdf]

ROC Curve - Random Forest (AUC = 1.0000)

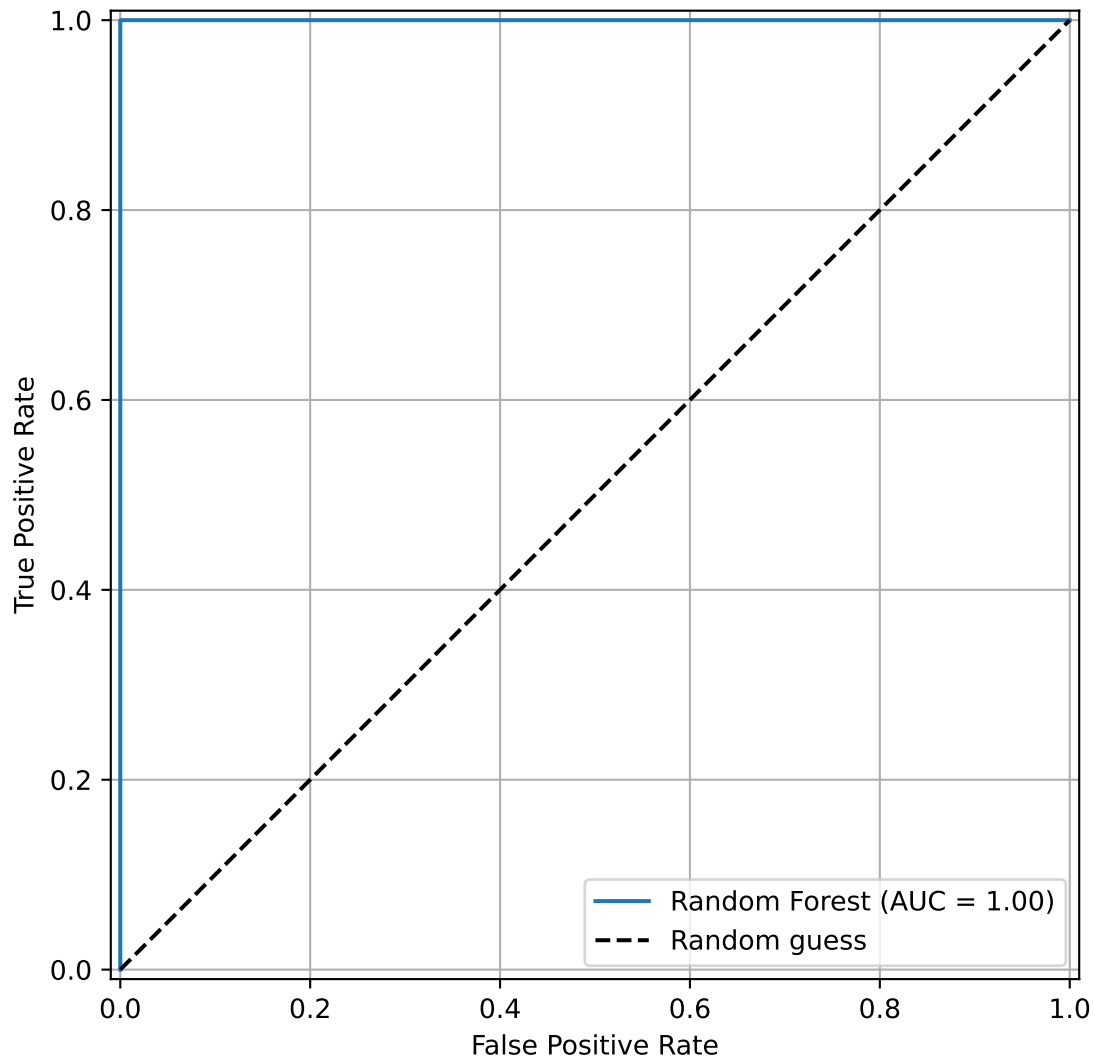

Supplement: Supplementary file 5 [file DataSheet5.zip › Supplementary_Data_Evaluation_Validation/Cancer_NonCancer/random_forest_roc.pdf]

ROC Curve - SVM (AUC = 1.0000)

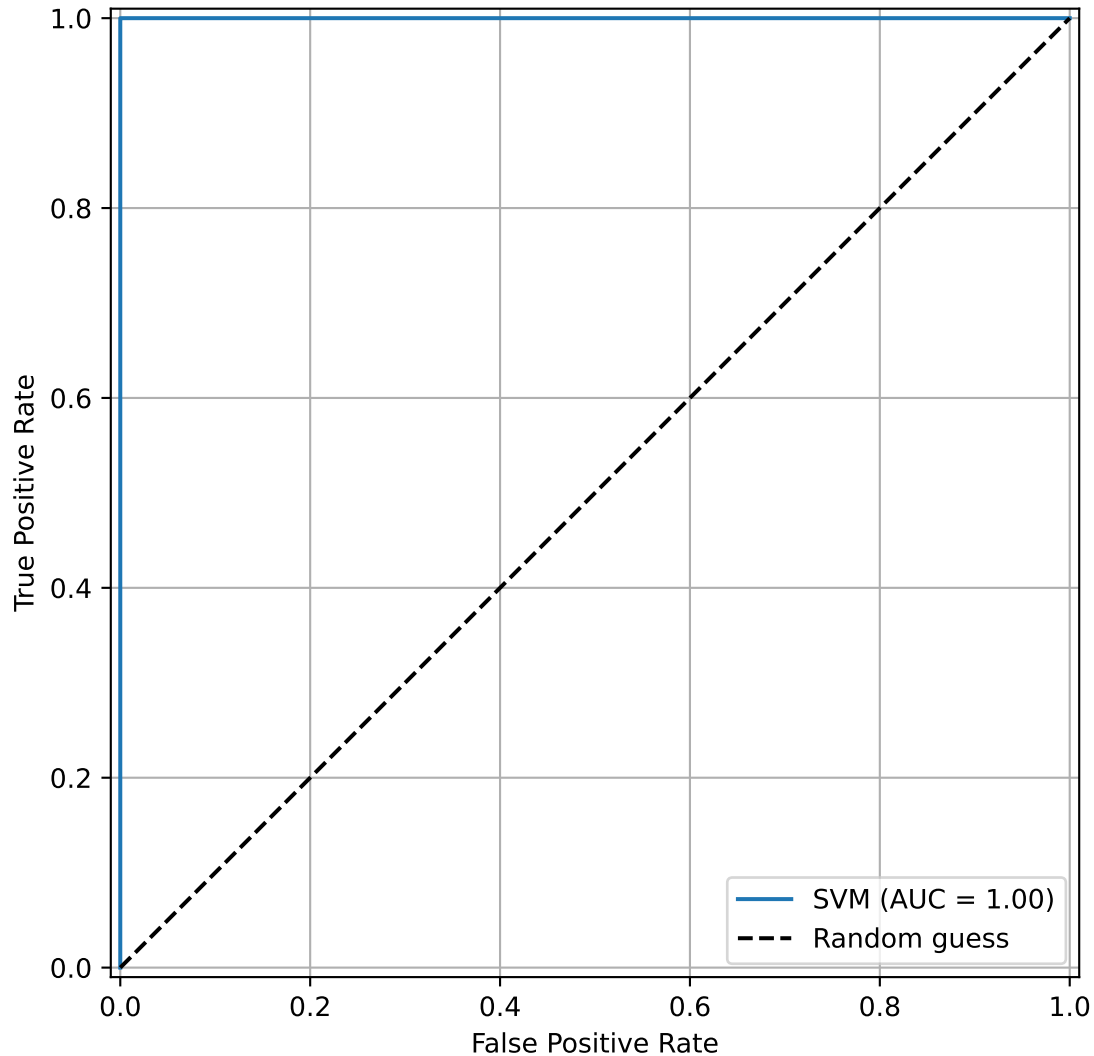

Supplement: Supplementary file 5 [file DataSheet5.zip › Supplementary_Data_Evaluation_Validation/Cancer_NonCancer/svm_roc.pdf]

ROC Curve - ANN

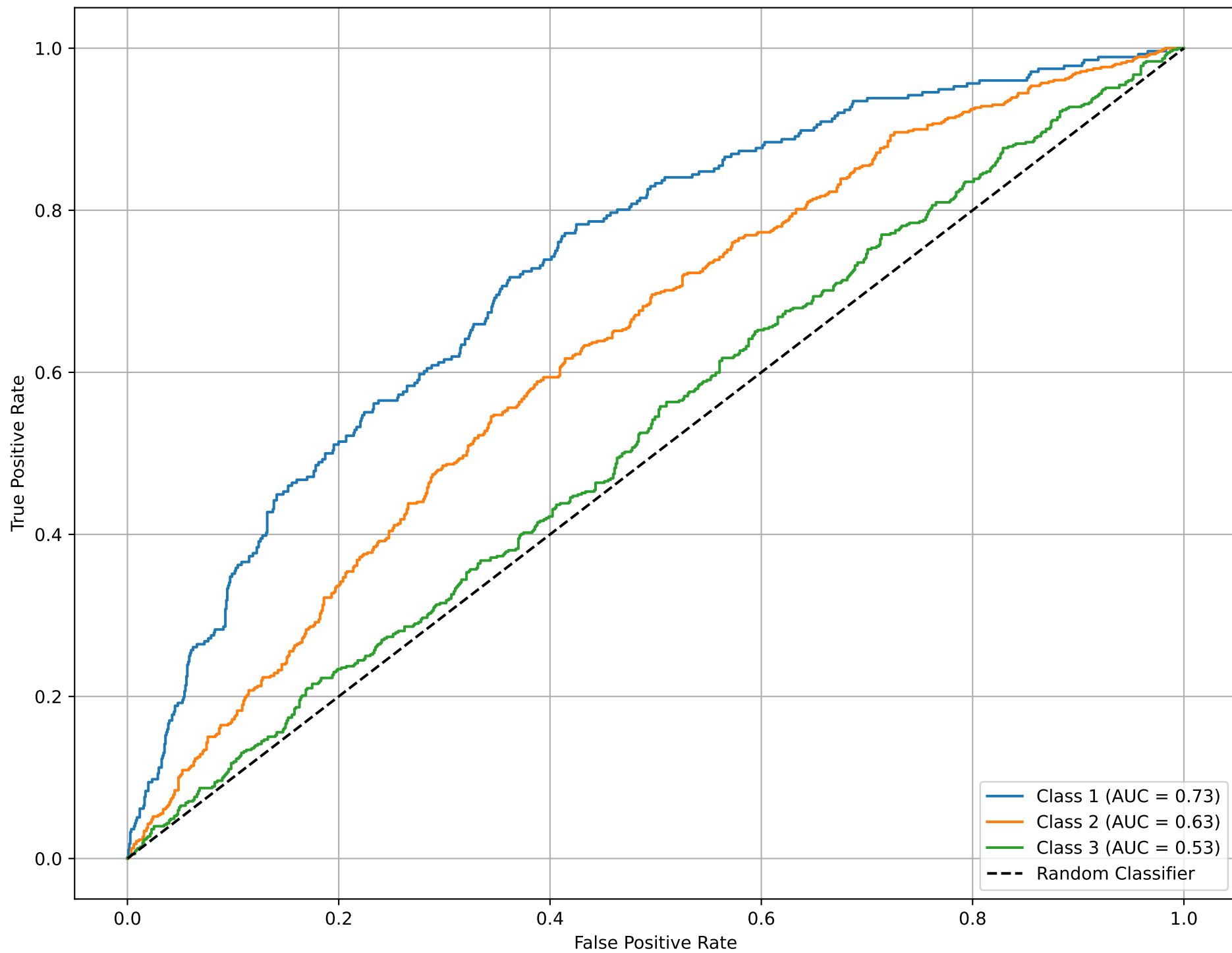

Supplement: Supplementary file 5 [file DataSheet5.zip › Supplementary_Data_Evaluation_Validation/IndependentData_Selleck_Angiogenesis_Cardio_Viral/roc_curves_ANN.pdf]

ROC Curve - Decision Tree

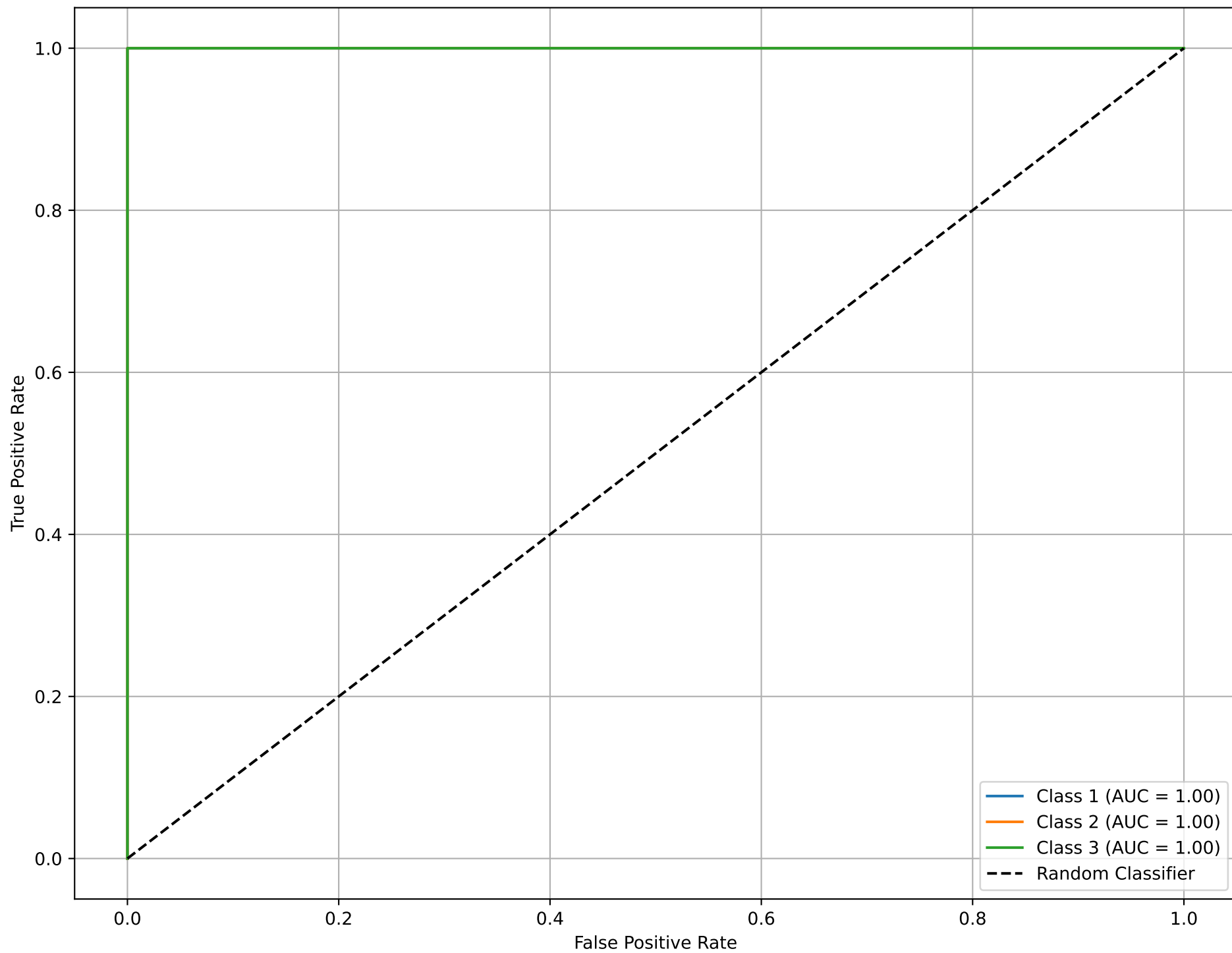

Supplement: Supplementary file 5 [file DataSheet5.zip › Supplementary_Data_Evaluation_Validation/IndependentData_Selleck_Angiogenesis_Cardio_Viral/roc_curves_Decision_Tree.pdf]

ROC Curve - Logistic Regression

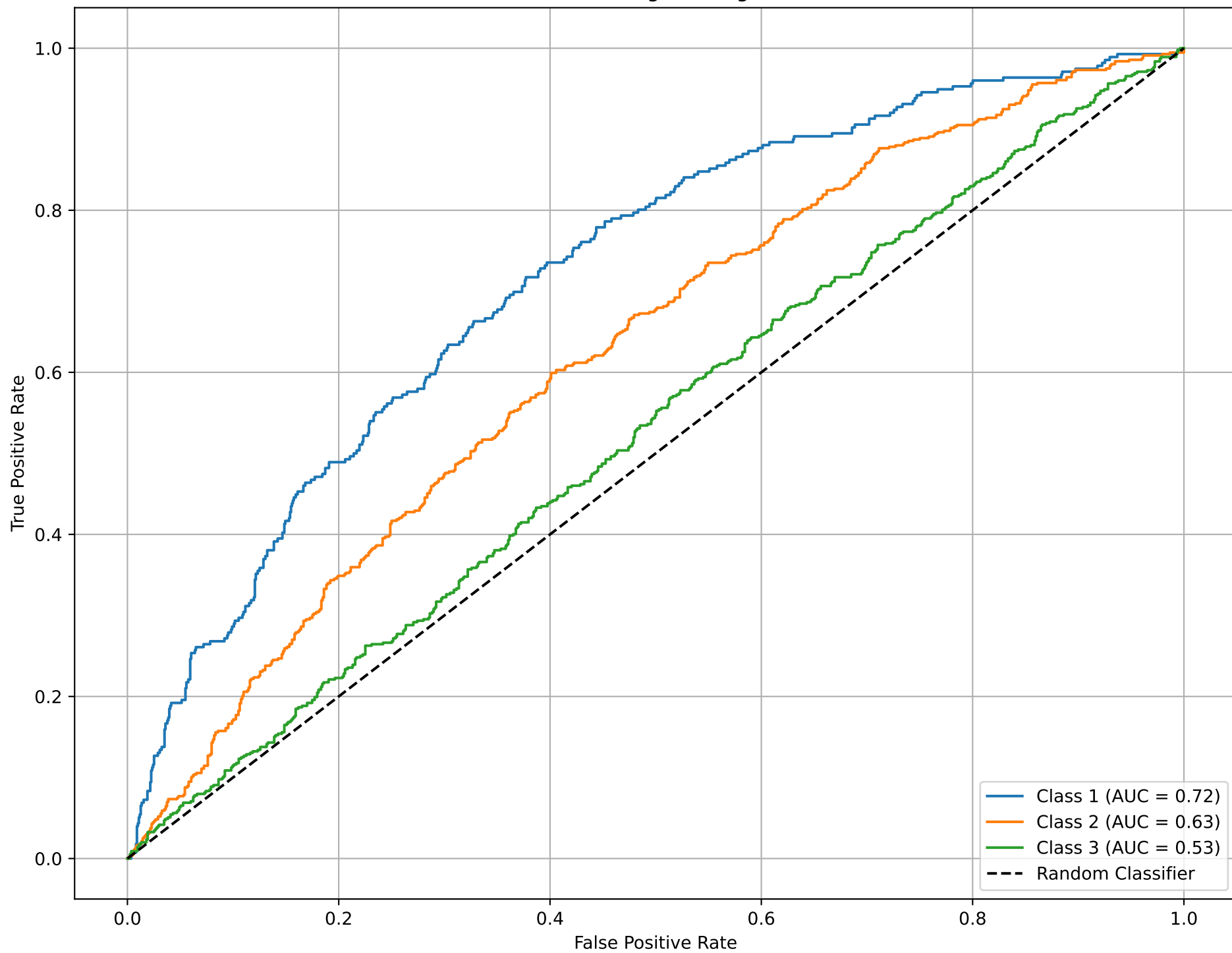

Supplement: Supplementary file 5 [file DataSheet5.zip › Supplementary_Data_Evaluation_Validation/IndependentData_Selleck_Angiogenesis_Cardio_Viral/roc_curves_Logistic_Regression.pdf]

ROC Curve - Random Forest

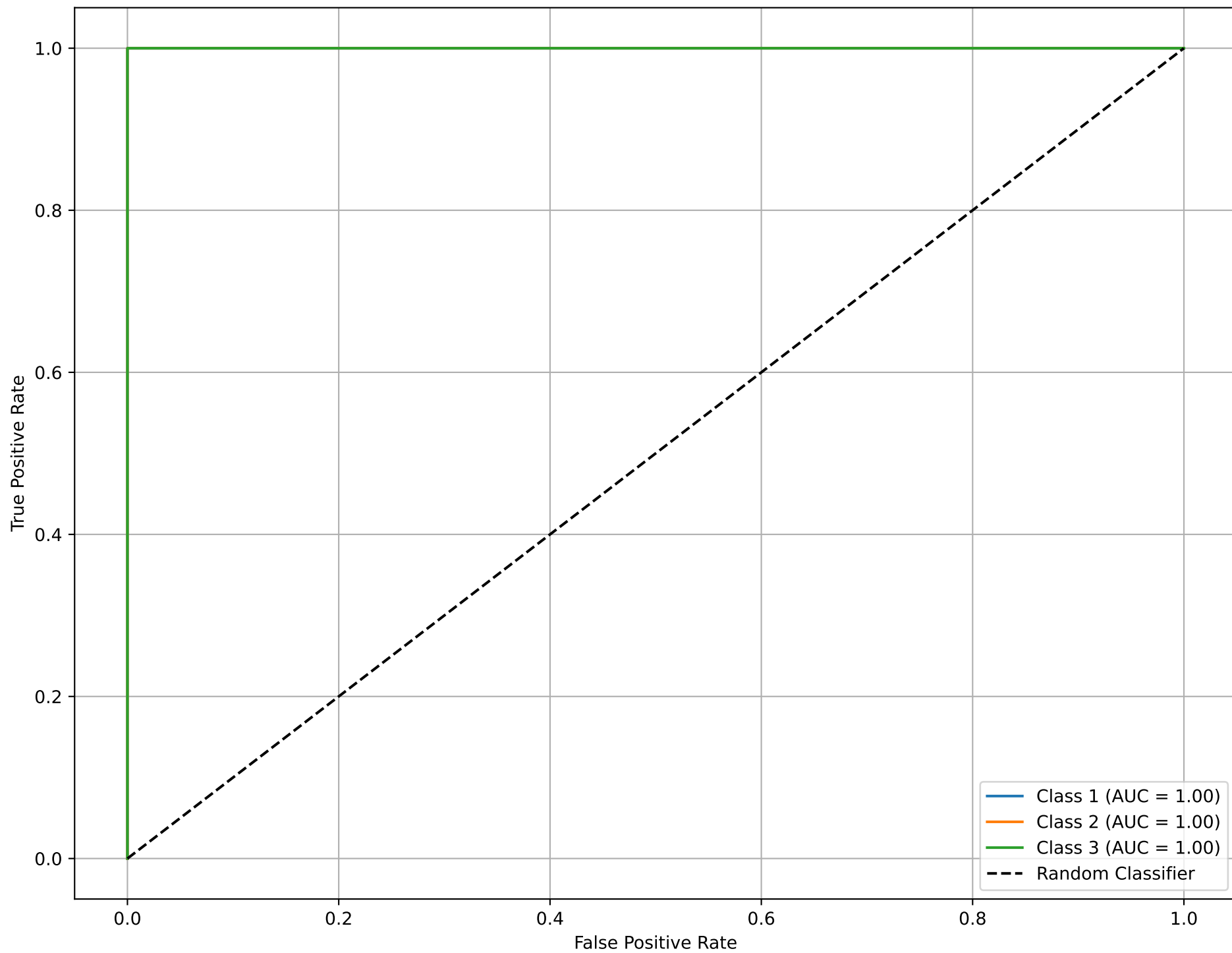

Supplement: Supplementary file 5 [file DataSheet5.zip › Supplementary_Data_Evaluation_Validation/IndependentData_Selleck_Angiogenesis_Cardio_Viral/roc_curves_Random_Forest.pdf]

ROC Curve - SVM

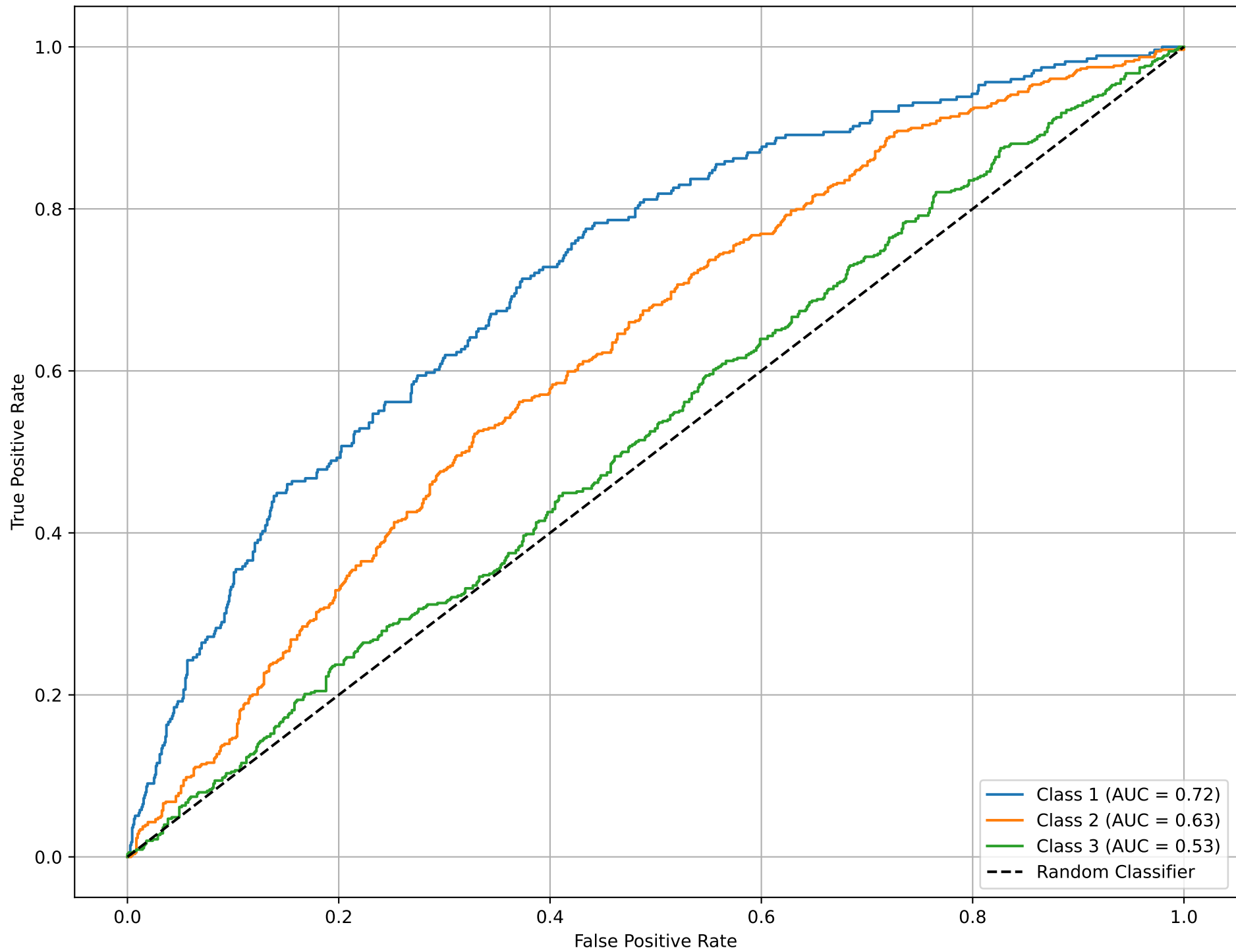

Supplement: Supplementary file 5 [file DataSheet5.zip › Supplementary_Data_Evaluation_Validation/IndependentData_Selleck_Angiogenesis_Cardio_Viral/roc_curves_SVM.pdf]

ROC Curve for ANN (One-vs-Rest)

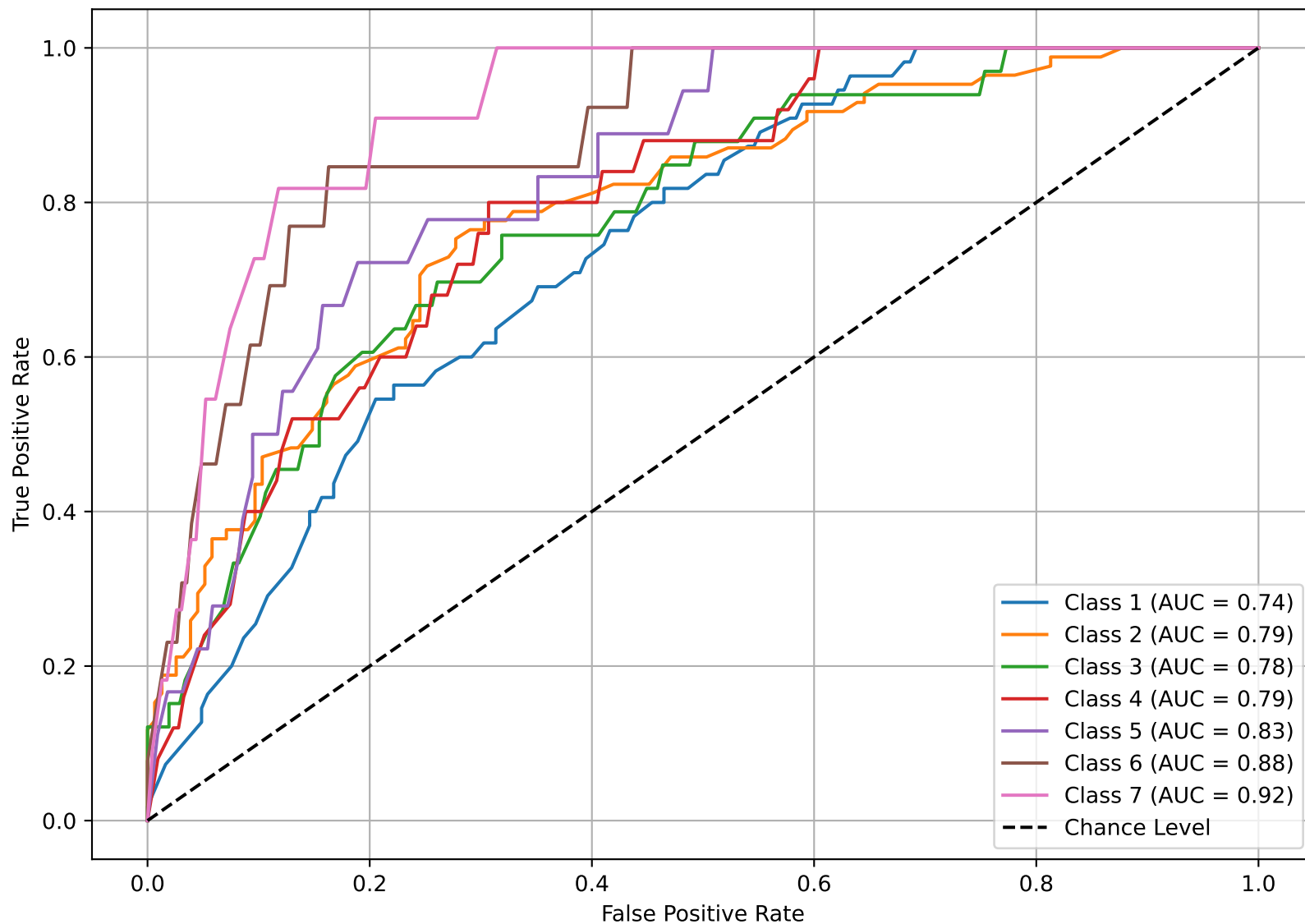

Supplement: Supplementary file 5 [file DataSheet5.zip › Supplementary_Data_Evaluation_Validation/MultiClass/roc_ann.pdf]

ROC Curve for Decision Tree (One-vs-Rest)

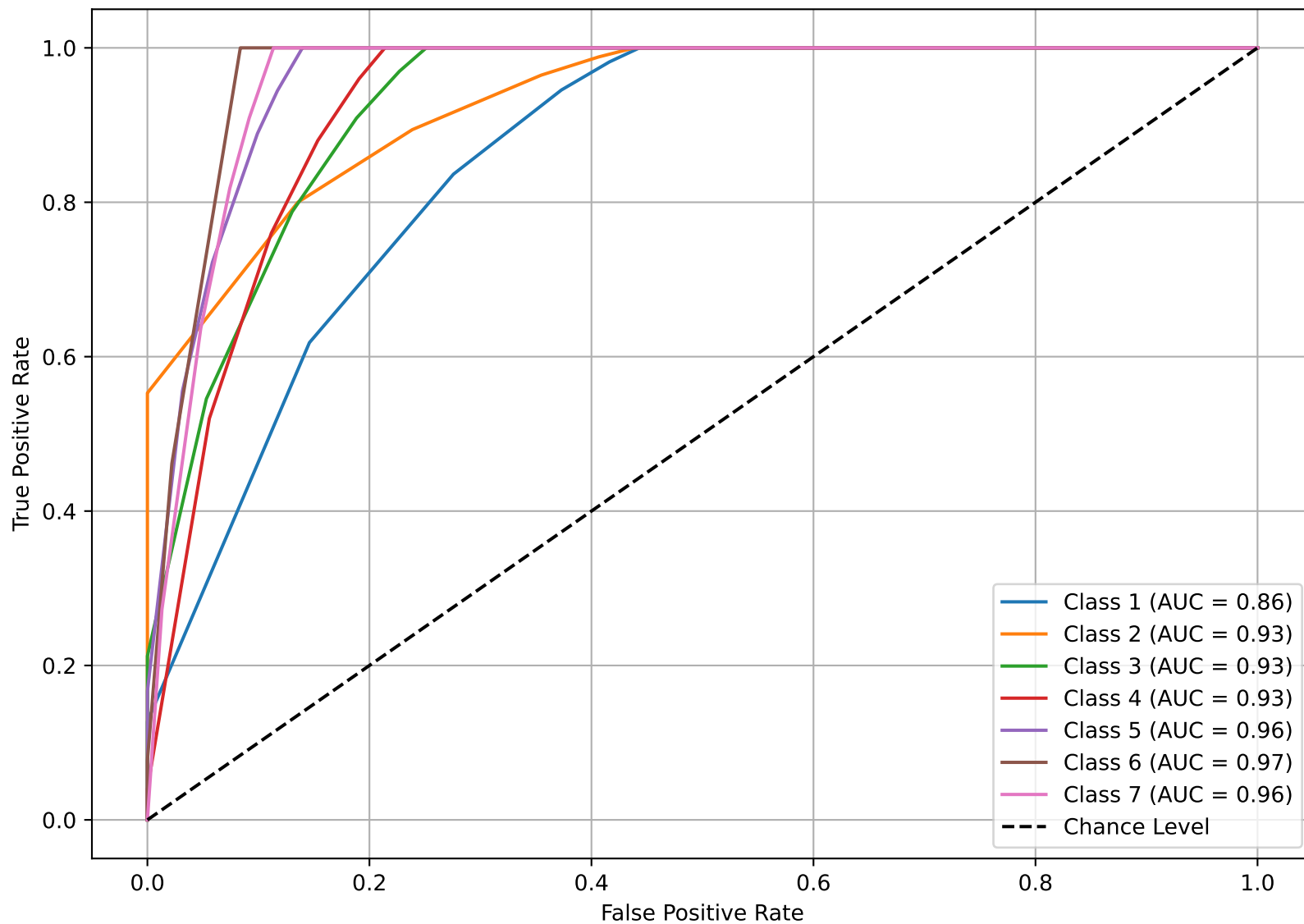

Supplement: Supplementary file 5 [file DataSheet5.zip › Supplementary_Data_Evaluation_Validation/MultiClass/roc_decision_tree.pdf]

ROC Curve for Logistic Regression (One-vs-Rest)

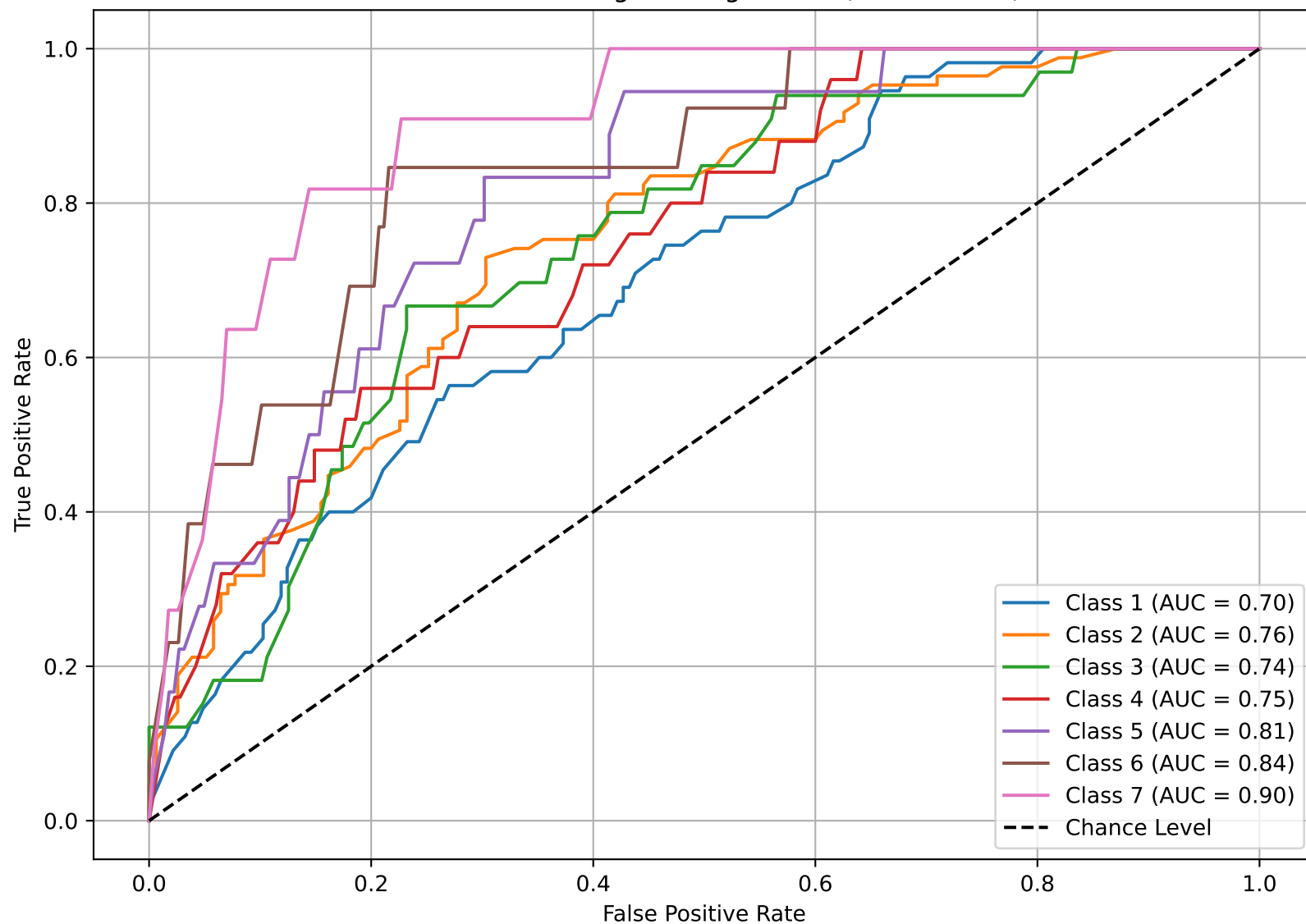

Supplement: Supplementary file 5 [file DataSheet5.zip › Supplementary_Data_Evaluation_Validation/MultiClass/roc_logistic_regression.pdf]

ROC Curve for Random Forest (One-vs-Rest)

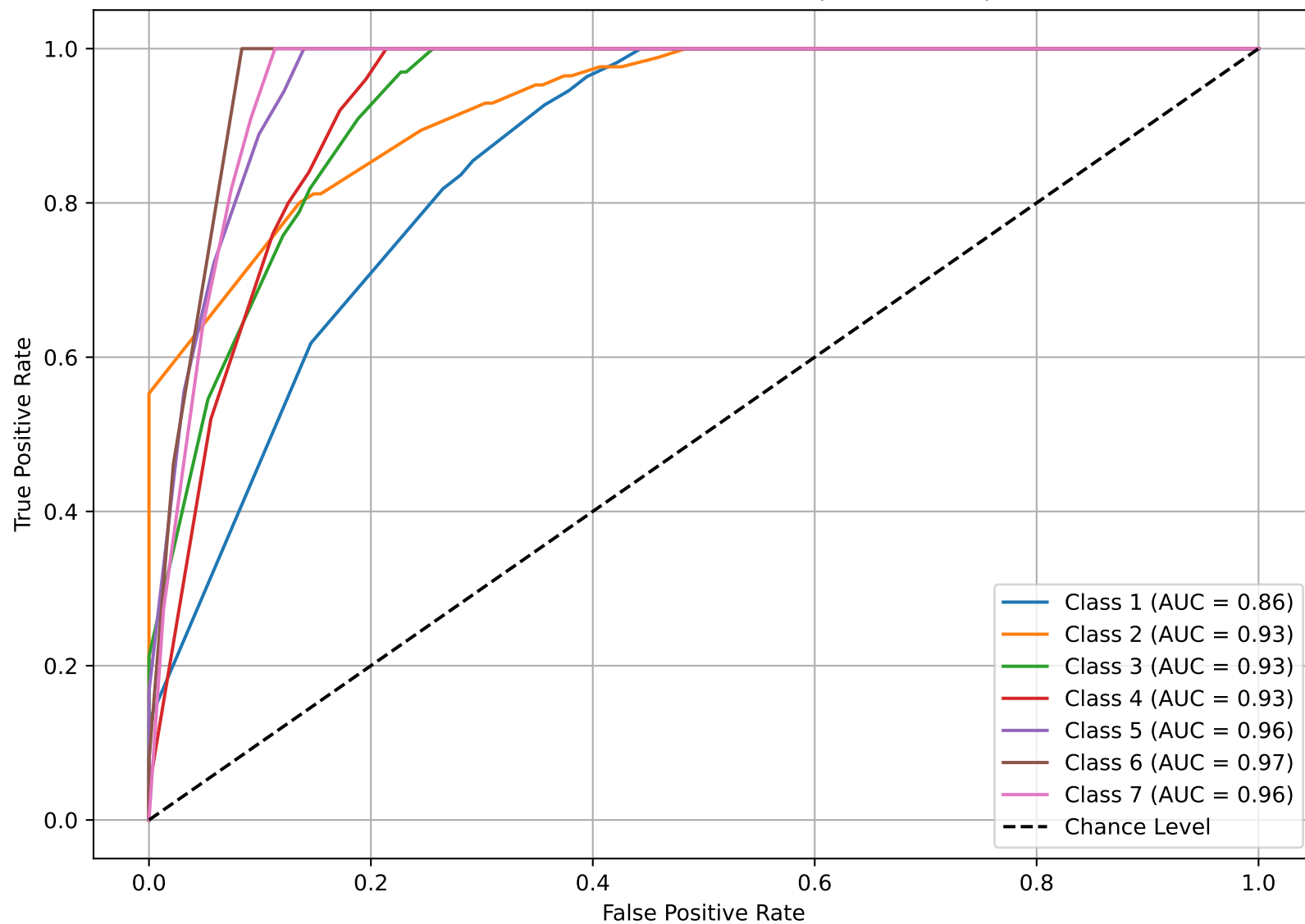

Supplement: Supplementary file 5 [file DataSheet5.zip › Supplementary_Data_Evaluation_Validation/MultiClass/roc_random_forest.pdf]

ROC Curve for SVM (One-vs-Rest)

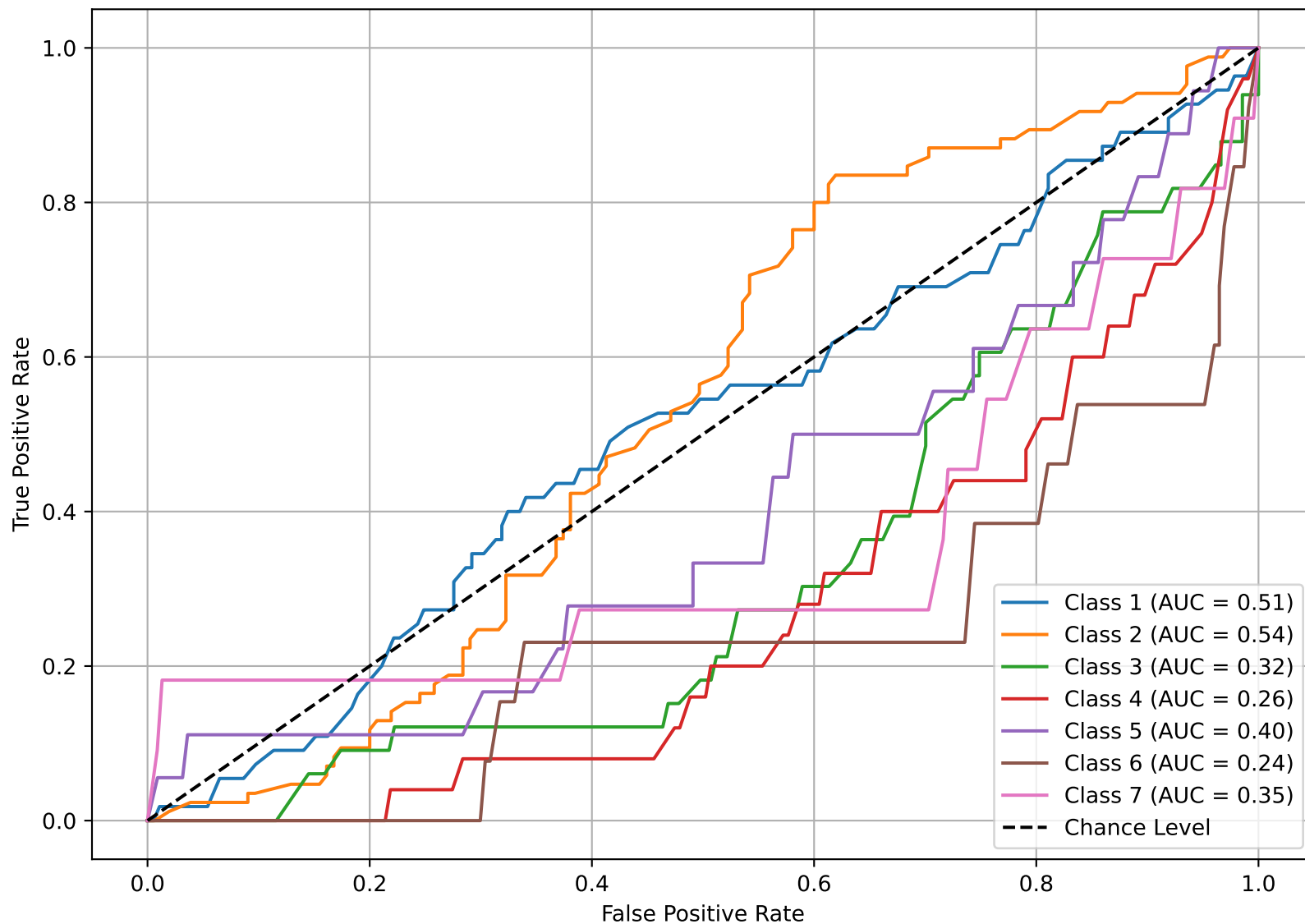

Supplement: Supplementary file 5 [file DataSheet5.zip › Supplementary_Data_Evaluation_Validation/MultiClass/roc_svm.pdf]
