# Supplementary material for: A network-driven computational framework for identifying FDA-approved drug repurposing across heterogeneous brain cancers
Source: Front Mol Biosci. 2026 Feb 17;13:1768081. doi: 10.3389/fmolb.2026.1768081 (PMC12953378; doi:10.3389/fmolb.2026.1768081)
Supplement: Supplementary file 5 [file DataSheet5.zip › Supplementary_Data_Evaluation_Validation/IndependentData_Selleck_Angiogenesis_Cardio_Viral/roc_curves_all_models.pdf]

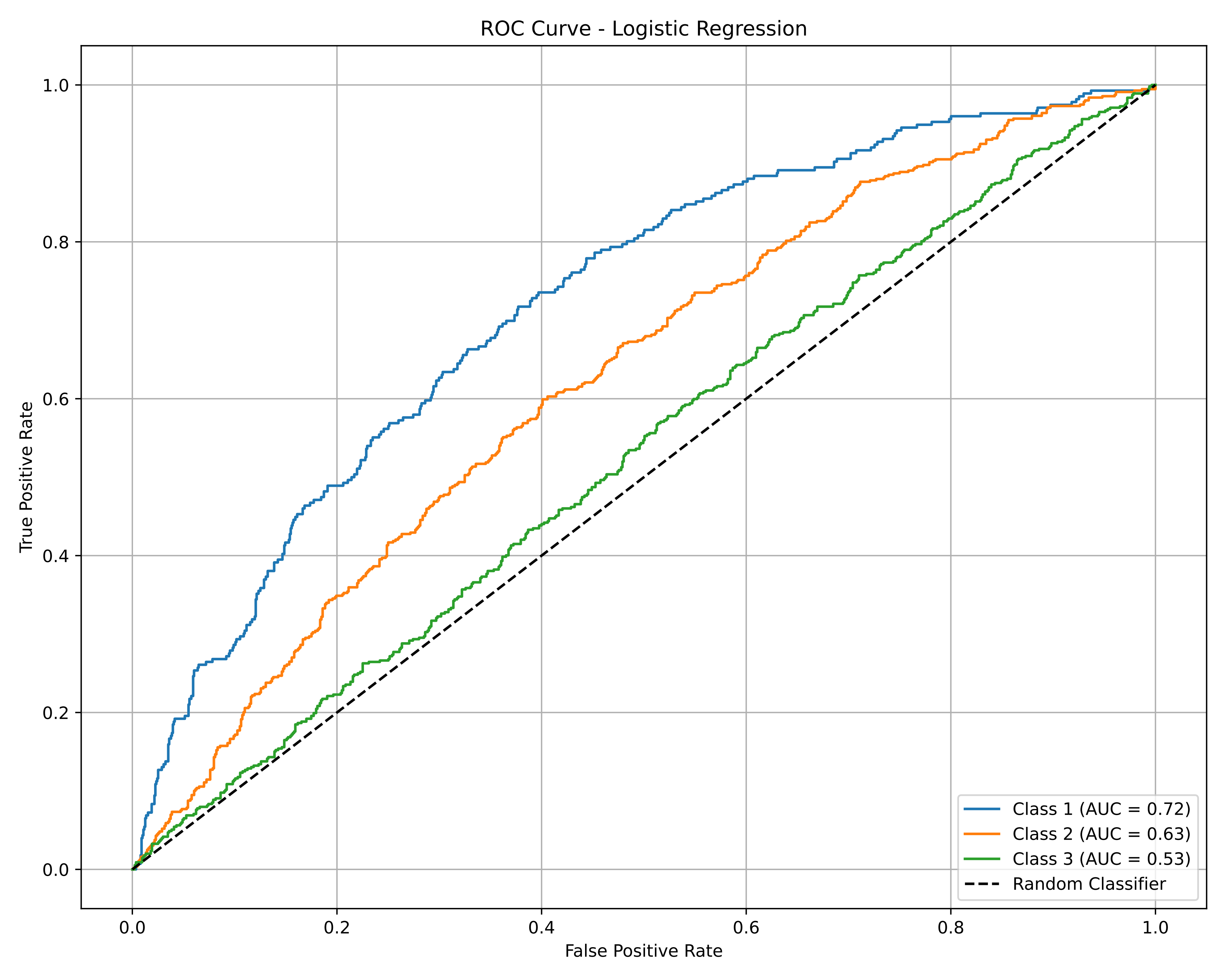
ROC Curve - Logistic Regression
True Positive Rate
False Positive Rate
0.0
0.2
0.4
0.6
0.8
1.0
0.0
0.2
0.4
0.6
0.8
1.0
Class 1 (AUC = 0.72)
Class 2 (AUC = 0.63)
Class 3 (AUC = 0.53)
Random Classifier

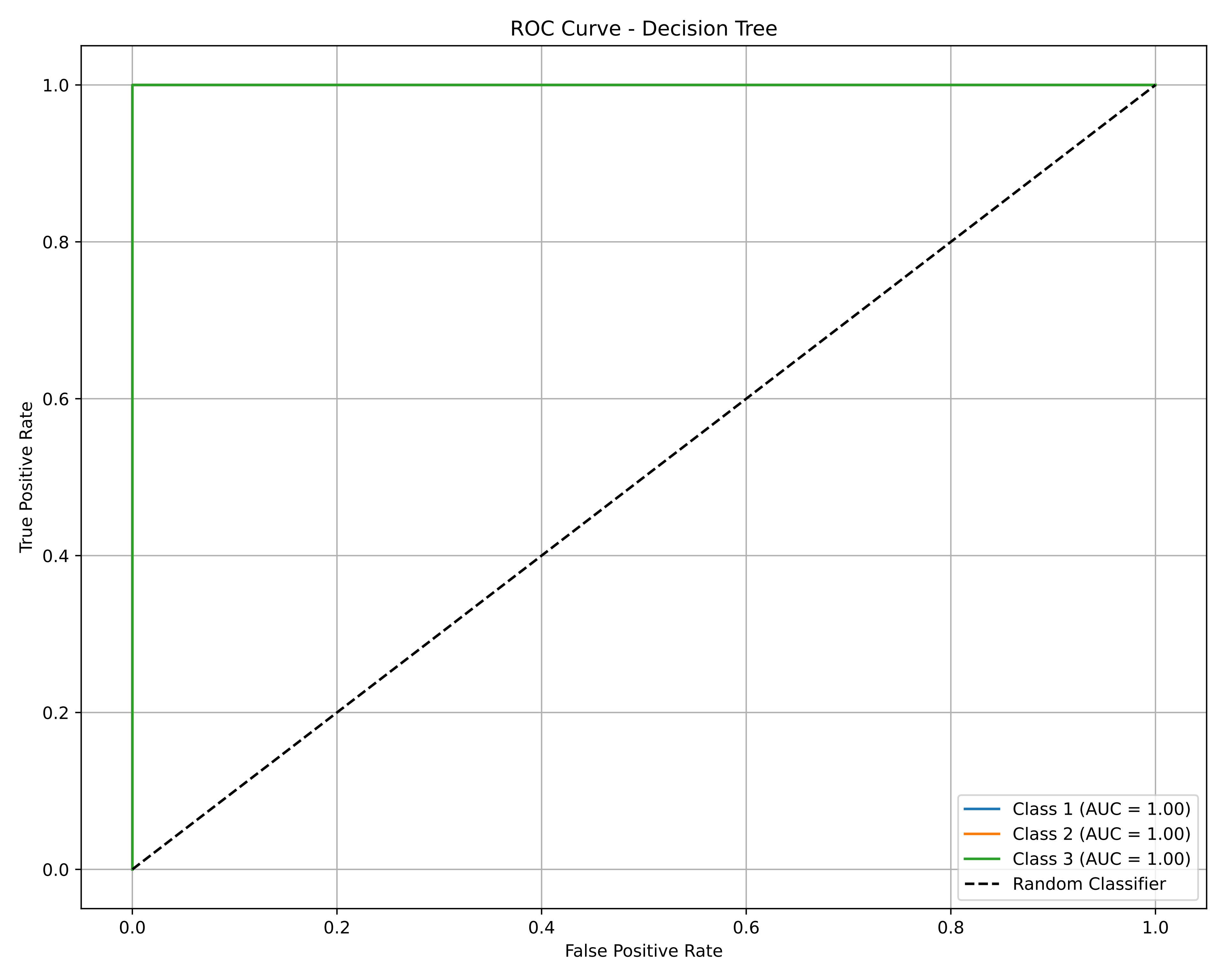
ROC Curve - Decision Tree
True Positive Rate
False Positive Rate
0.0
0.2
0.4
0.6
0.8
1.0
Class 1 (AUC = 1.00)
Class 2 (AUC = 1.00)
Class 3 (AUC = 1.00)
Random Classifier

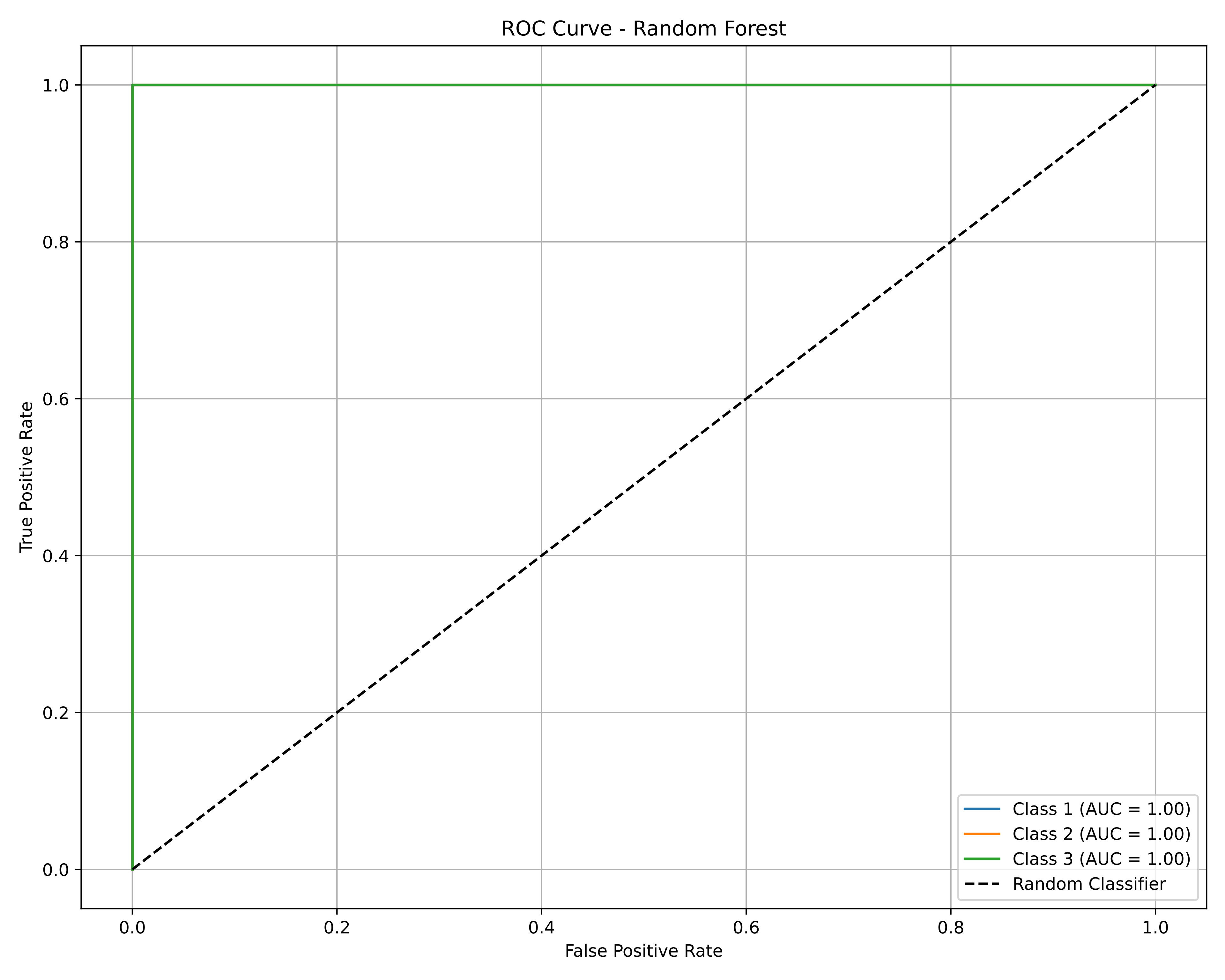

ROC Curve - Random Forest
True Positive Rate
False Positive Rate
0.0
0.2
0.4
0.6
0.8
1.0
Class 1 (AUC = 1.00)
Class 2 (AUC = 1.00)
Class 3 (AUC = 1.00)
Random Classifier

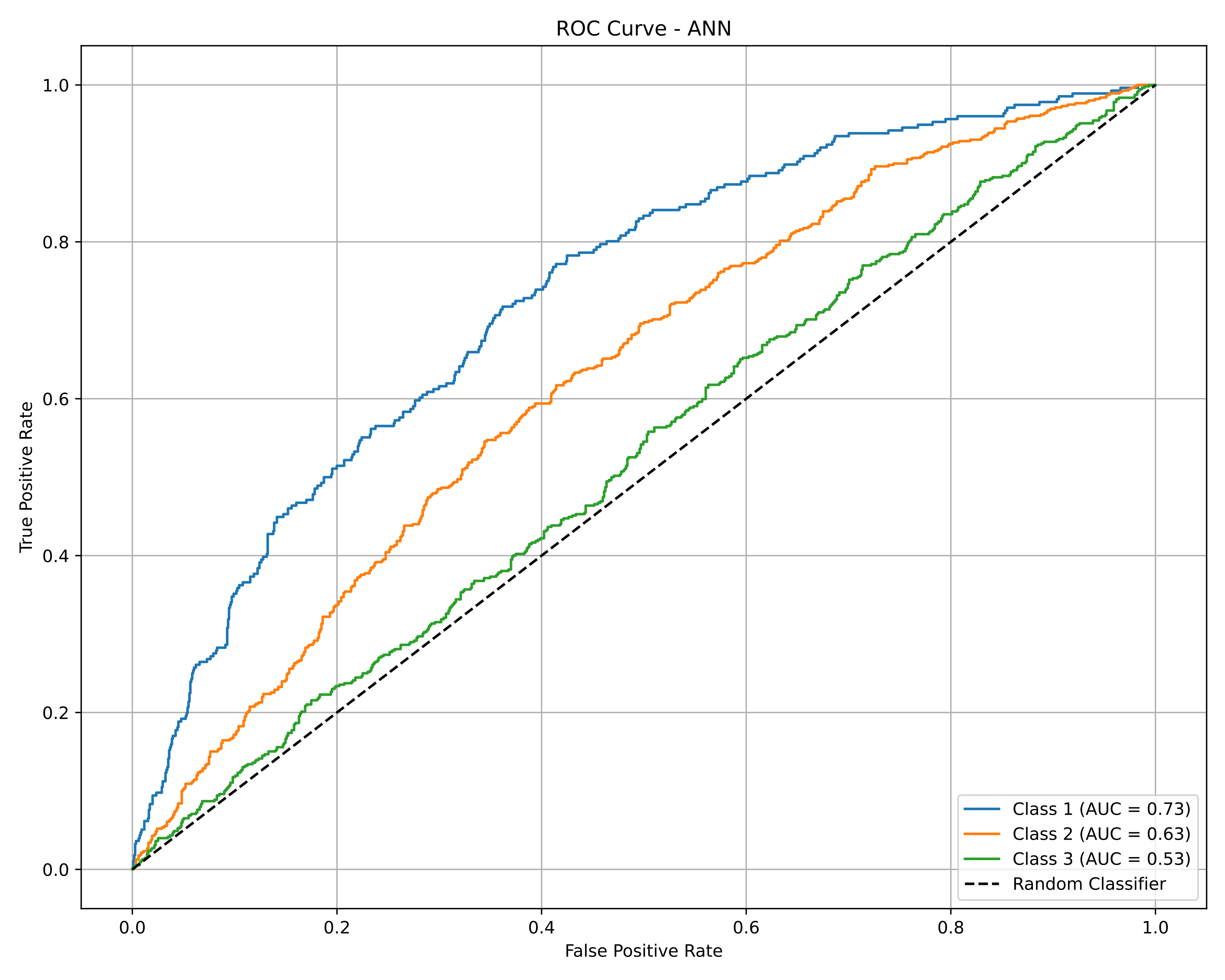
ROC Curve - ANN
True Positive Rate
False Positive Rate
0.0
0.2
0.4
0.6
0.8
1.0
Class 1 (AUC = 0.73)
Class 2 (AUC = 0.63)
Class 3 (AUC = 0.53)
Random Classifier

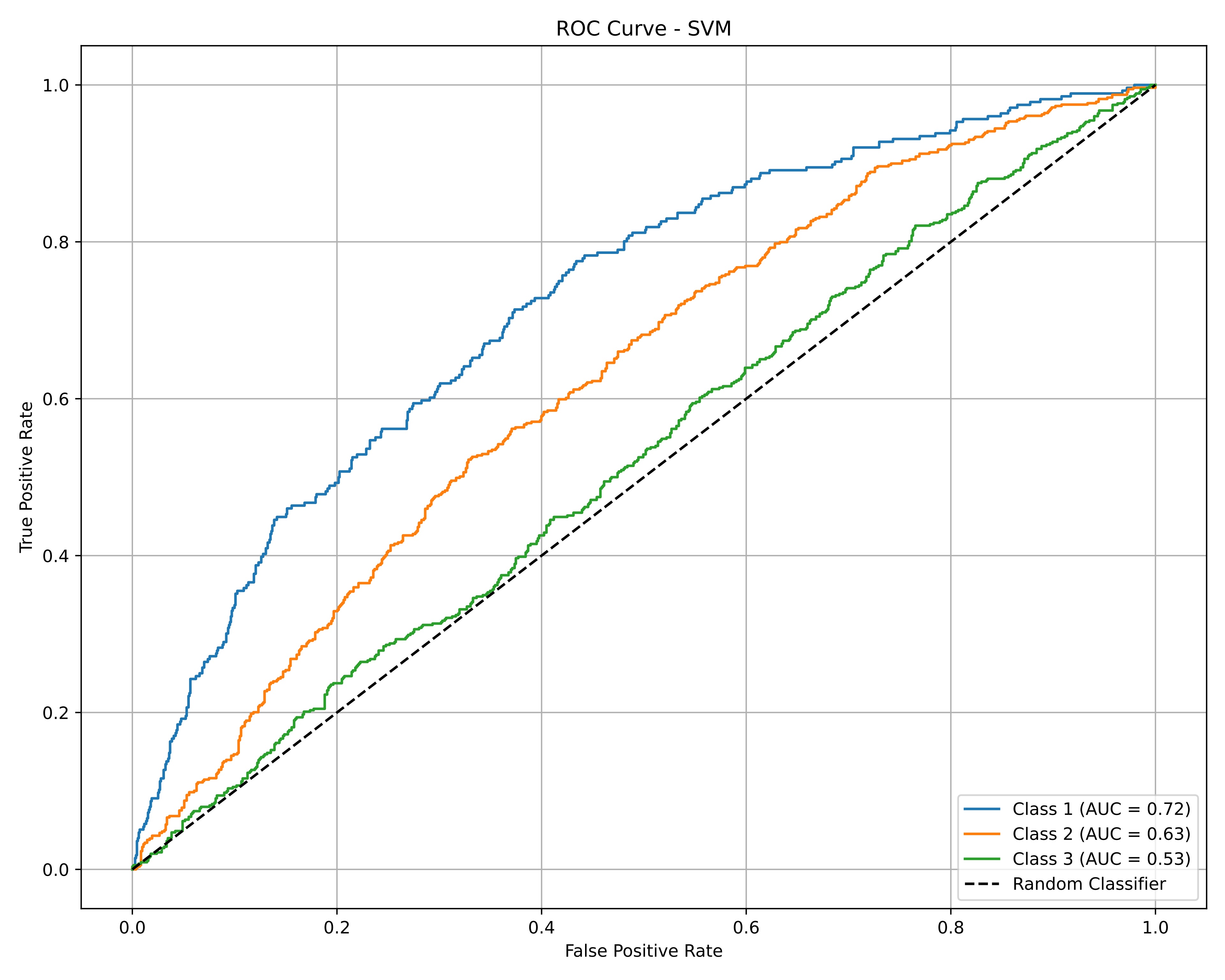
ROC Curve - SVM
True Positive Rate
False Positive Rate
0.0
0.2
0.4
0.6
0.8
1.0
Class 1 (AUC = 0.72)
Class 2 (AUC = 0.63)
Class 3 (AUC = 0.53)
Random Classifier
